# Altered Protein Interactions of the Endogenous Interactome of PTPIP51 towards MAPK Signaling

**DOI:** 10.3390/biom7030055

**Published:** 2017-07-21

**Authors:** Alexander Brobeil, Rajaa Chehab, Eric Dietel, Stefan Gattenlöhner, Monika Wimmer

**Affiliations:** 1 Institute of Anatomy and Cell Biology, Justus-Liebig-University, 35392 Giessen, Germany; rajaa.chehab@anatomie.med.uni-giessen.de (R.C.); eric.dietel@med.uni-giessen.de (E.D.); monika.wimmer@anatomie.med.uni-giessen.de (M.W.); 2 Institute of Pathology, Justus-Liebig-University, 35392 Giessen, Germany; stefan.gattenloehner@patho.med.uni-giessen.de

**Keywords:** PTPIP51, LDC-3, protein–protein interaction, protein complex, scaffold protein

## Abstract

Protein–protein interactions play a pivotal role in normal cellular functions as well as in carcinogenesis. The protein–protein interactions form functional clusters during signal transduction. To elucidate the fine calibration of the protein–protein interactions of protein tyrosine phosphatase interacting protein 51 (PTPIP51) a small molecule drug, namely LDC-3, directly targeting PTPIP51 is now available. Therefore, LDC-3 allows for the studying of the regulation of the endogenous interactome by modulating PTPIP51 binding capacity. Small interfering ribonucleic acid (siRNA) experiments show that the modification in PTPIP51 binding capacity is induced by LDC-3. Application of LDC-3 annuls the known regulatory phosphorylation mechanisms for PTPIP51 and consequently, significantly alters the assembly of the PTPIP51 associated protein complexes. The treatment of human keratinocytes (HaCaT cells) with LDC-3 induces an altered protein–protein interaction profile of the endogenous interactome of PTPIP51. In addition, LDC-3 stabilizes PTPIP51 within a mitogen activated protein kinase (MAPK) complex composed of Raf-1 and the scaffold protein 14-3-3, independent of the phosphorylation status of PTPIP51. Of note, under LDC-3 treatment the regulatory function of the PTP1B on PTPIP51 fails to impact the PTPIP51 interaction characteristics, as reported for the HaCaT cell line. In summary, LDC-3 gives the unique opportunity to directly modulate PTPIP51 in malignant cells, thus targeting potential dysregulated signal transduction pathways such as the MAPK cascade. The provided data give critical insights in the therapeutic potential of PTPIP51 protein interactions and thus are basic for possible targeted therapy regimens.

## 1. Introduction

Protein tyrosine phosphatase interacting protein 51 (PTPIP51) was identified in various protein complexes fulfilling opposite cellular functions, namely proliferation, differentiation and apoptosis [1,2]. Meanwhile, a growing number of PTPIP51 interaction partners have been described. Recent studies specified the identified interaction partners including kinases and phosphatases, linker proteins such as 14-3-3, cell receptors (epidermal growth factor receptor (EGFR) and insulin receptor (IR)) [3,4,5,6], transcription factors (nuclear factor κB [NFκB]) [7], transport associated proteins (neurophysin) [8], mitotic associated proteins (CGI-99 and Nuf2) [3], diacylglycerol kinase α (DGKα), a modulator of the second messenger diacylglycerol [9], and regulators of Ca^2+^ homeostasis (vesicle associated protein B (VAPB)) [10,11,12].

Many tumors display an altered PTPIP51 protein expression and an anomalous subcellular distribution [13,14] as has been observed in the cells of basal cell carcinoma and squamous cell carcinoma [15]. Additionally, in malignant blasts of acute myeloid leukemia (AML) samples PTPIP51 is expressed in contrast to cells of healthy bone marrow specimen with no PTPIP51 protein expression [16,17]. In AML blasts the interaction of PTPIP51 with the mitogen activated protein kinase (MAPK) pathway is inhibited due to its high Tyr176 phosphorylation level [16].

The MAPK pathway modulation is arbitrated by the scaffold protein family 14-3-3 [18]. When bound to 14-3-3 proteins, then PTPIP51 is empowered to modulate the MAPK pathway [18]. To avoid an over-activation of the MAPK pathway, the formation of PTPIP51 protein complexes with 14-3-3 and Raf-1 is tightly regulated by distinct kinases and phosphatases [4,9,16]. When PTPIP51 is phosphorylated at its Tyr176 residue, the ternary complex of PTPIP51, 14-3-3 and Raf-1 dissociates into a PTPIP51/14-3-3 complex and Raf-1 [9]. The Tyr176 phosphorylation of PTPIP51 is mediated by the EGFR and c-Src and antagonized by protein tyrosine phosphatase 1B (PTP1B) [4]. Besides its regulatory function, the Tyr176 phosphorylation of PTPIP51 also alters subcellular localization [4]. When Tyr176 is phosphorylated, PTPIP51 localizes to the mitotic spindle apparatus interacting with the microtubular system [3]. CGI-99 and Nuf-2 were identified as interaction partners of PTPIP51 forming the mitotic protein complex of PTPIP51 [3]. Of note, this mitotic PTPIP51 protein complex was also traced in interphase cells [3]. Besides its involvement in the MAPK mediated cell survival and in the mitotic process, PTPIP51 is also linked to apoptosis [19]. In addition, PTPIP51 impacts the contact of mitochondria to the endoplasmic reticulum (ER) by interacting with VAPB and regulates normal calcium homeostasis [10,11].

Yet, up until now the exact regulation of the interactions and relocation of PTPIP51 is not fully understood. Recently, small molecule compounds have gained the spotlight, especially in cancer research and for therapeutical applications [20]. LDC-3 is a newly synthesized small molecule that targets the cytoplasmic dynein when used in nanomolar concentrations [21]. Yet, application of the small molecule in µmolar concentration resulted in an interaction with PTPIP51 [21], an off-target, and thereby modulates its function. Studying the effects of LDC-3 on the interactome has the potential to give deep and detailed insights into the formation and regulation of PTPIP51 protein complexes.

This new compound has enabled us to initiate such a study using the PTPIP51 expressing HaCaT cell line. This non-tumorigenic spontaneously immortalized keratinocyte cell line was extensively investigated for the functional regulation and interaction patterns of PTPIP51. Thus, this cell line is optimally suited to a more detailed protein complex investigation using the small molecule compound LDC-3. HaCaT cells were exposed to LDC-3 in a dose and time dependent manner. The protein–protein interactions were evaluated using the Duolink proximity ligation assay (DPLA), which provides real time in situ interaction data suitable for quantification. By combing LDC-3 with the in situ interaction-profiling assay—DPLA—this study gives critical new insights in the regulation of the endogenous interactome of PTPIP51 in normal non-tumorigenic cells. Analysis of these protein networks by LDC-3 provides a drugable alteration in the PTPIP51 interactions, thereby, interacting and activating the MAPK pathway. This information might be useful for the generation of a therapeutic strategy targeting the PTPIP51 interactome in dysregulated, disease-relevant pathways.

## 2. Results

### 2.1. LDC-3 Effects on the Activation Status of the MAPK by Immunoblotting

**Figure 1:**
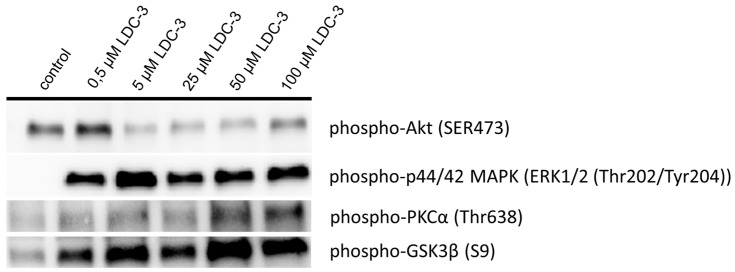
Immunoblotting of p42/p44-mitogen activated protein kinase (MAPK), Akt and cyclin-dependent kinase 1 (CDK1) activity. HaCaT cells were treated with increased dose of LDC-3 (*n* = 3). The activation status of p42/p44-MAPK, Akt, protein kinase C α (PKCα) and glycogensynthase kinase 3β (GSK3β) were evaluated using specific antibody raised against activating phosphotyrosine/phosphothreonine residues (p42/p44-MAPK), activating phosphoserine residue (Akt), activating phosphothreonine residue (PKCα) or inhibiting phosphoserine residue (GSK3β). The immunoblots were normalized to the stain-free blot shown in the supplementary information (Supplementary Materials Figure S6A).

As revealed by immunoblotting experiment applying LDC-3 in doses of 5 µM and 50 µM for incubation times of 12 h to HaCaT cells, increased the p42/p44-MAPK (Erk1/2) phosphorylation and decreased the Ser473 phosphorylated Akt (Figure 1).

To get insights in the regulation of the ER interaction with mitochondria, we investigated the activation status of the glycogen synthase kinase 3 β (GSK3β) and protein kinase Cα (PKCα) by immunoblotting (Figure 1). Here, LDC-3 effects on PTPIP51 induced a higher phosphorylation level at the Ser9 residue of GSK3β in relation to the level seen in cells of the control group, which signified its inactivation (Figure 1). PKCα was phosphorylated at its threonine 638 residue as compared to the control group, indicating the activation of the kinase (Figure 1).

### 2.2. LDC-3 Binds Specific to PTPIP51 Tested by siRNA Knock down Experiments

**Figure 2:**
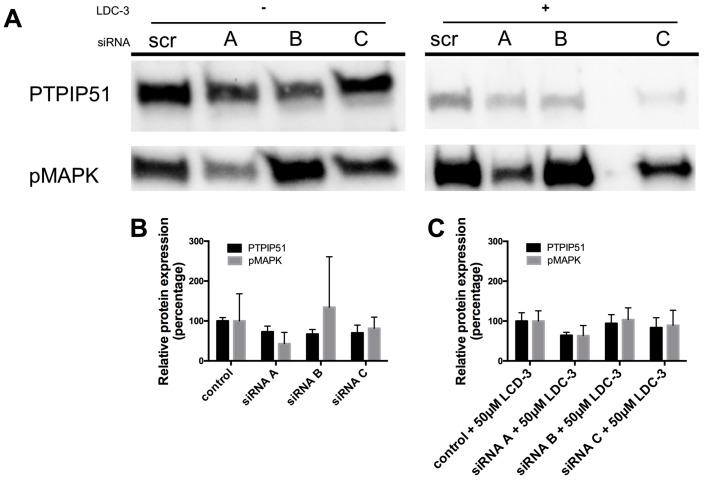
Small interfering ribonucleic acid (siRNA) experiments verifying the specific binding of LDC-3. (**A**) Cell lysate of all siRNA constructs (*n* = 3) were probed with the antibody against protein tyrosine phosphatase interacting protein 51 (PTPIP51) and p42/p44-MAPK (Erk1/2). The lysates of the left panel lack LDC-3 treatment, the right panel displays siRNA experiments with additional LDC-3 treatment; (**B**) Graphical overview of the knock-down values without LDC-3 treatment; (**C**) Graphical overview of the knock-down values with LDC-3 treatment. The immunoblots were normalized to the stain-free blot shown in the supplementary information (Supplementary Materials Figure S6B).

Using three different small interfering ribonucleic acid (siRNA) constructs for PTPIP51, a specific knock down of total PTPIP51 protein could be traced for all three siRNA constructs A, B and C as compared to the scramble control (Figure 2). The knock-down directly influenced the MAPK pathway activity. For siRNA construct A and C a decrease in the phosphorylation level of the p42/p44-MAPK could be traced, whereas the application of the siRNA construct B slightly increased the p42/p44-MAPK phosphorylation (Figure 2A).

Applying LDC-3 to the scramble siRNA controls up-regulates p42/p44-MAPK phosphorylation, whereas adding LDC-3 to the siRNA construct A and C transfected cells had no effect on p42/p44-MAPK phosphorylation (Figure 2A). The siRNA construct B slightly increased the p42/p44-MAPK phosphorylation under LDC-3 treatment corresponding to the LDC-3 lacking siRNA experiment with construct B (Figure 2A). Figure 2B,C display the graphs for each knock-down experiment.

### 2.3. LDC-3 Effects on Mitochondrial Homeostasis and Cell Proliferation

**Figure 3:**
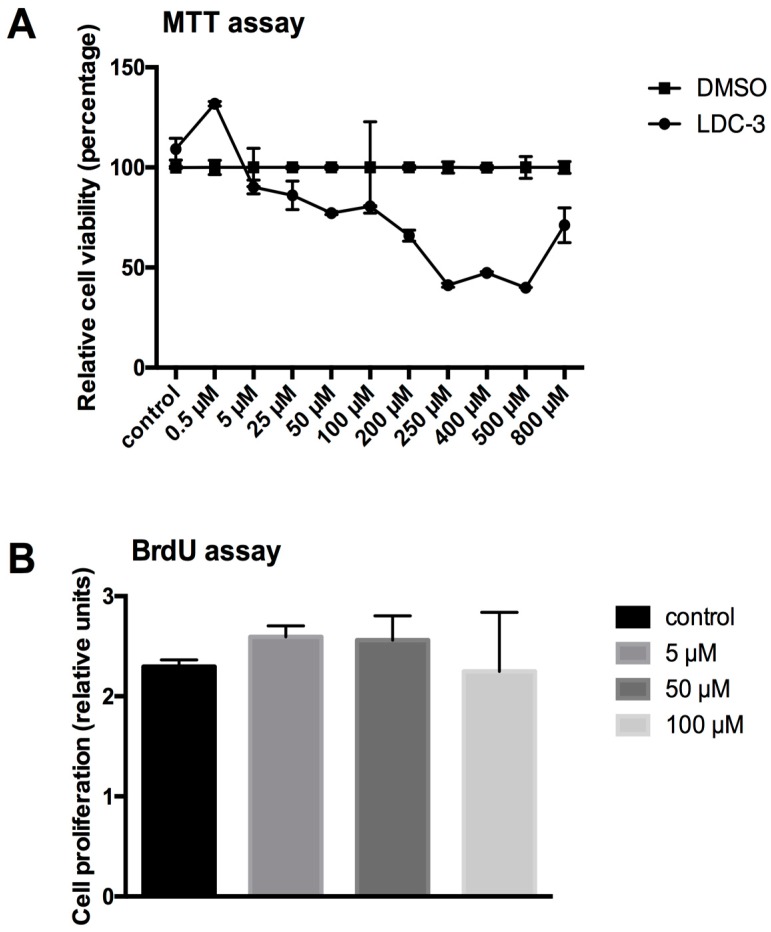
Cell viability (3-(4,5-dimethylthiazol-2-yl)-2,5-diphenyltetrazolium bromide (MTT)) and cell proliferation (bromodeoxyuridine (BrdU)) assay. (**A**) Cell viability of LDC-3 treated HaCaT cells for 24 h tested by an MTT assay. The values for LDC-3 treated cells were calculated as the percental quotient of the LDC-3 value and the DMSO value; (**B**) Relative proliferation rate of untreated HaCaT cells and cells treated for 12 h with 5 µM, 50 µM and 100 µM LDC-3. Mitotic nuclei were identified by incorporation of BrdU, which were immunocytochemically detected by horseradish peroxidase (HRP)-linked antibody. The transformation of the tetramethylbenzidine (TMB) substrate by the bound HRP was measured by reading the absorbance at 450 nm. No statistical differences could be revealed (*p* > 0.05). Dunnett’s multiple comparisons test.

The LDC-3 altered mitochondrial homeostasis was determined using a 3-(4,5-dimethylthiazol-2-yl)-2,5-diphenyltetrazolium bromide (MTT) assay kit. To exclude the toxic effect of dimethyl sulfoxide (DMSO), a second curve was established applying gradient amounts of DMSO comparable to the amount of effector added in rising concentrations to the test system. The values for LDC-3 treated cells were calculated as the percental quotient of the LDC-3 value and the DMSO value. As shown in Figure 3A, beginning at concentrations of 5 µM, there is a continuous decrease in the mitochondrial metabolism due to the added LDC-3. Lowest levels of metabolic rate were observed for 250 µM and 500 µM with a reduction to about 40% of control cells (Figure 3A). The structurally altered forms of LDC-3 (LDC-4 and LDC-9) had no effect on mitochondrial metabolic rate in the dose range of 0.5 µM to 200 µm (Supplementary Materials Figure S1).

In addition, the effect of LDC-3 on the HaCaT cell proliferation rate was determined by bromodeoxyuridine (BrdU) assays. LDC-3 treatment had no effect on cell proliferation (Figure 3B). Neither a decrease nor an increase in cellular proliferation of the HaCaT cells could be traced (Figure 3B).

### 2.4. LDC-3 Effects on the Protein Expression and the Phosphorylation Status of PTPIP51 Protein at Tyr176, Ser46 and Ser212 Residue

**Figure 4:**
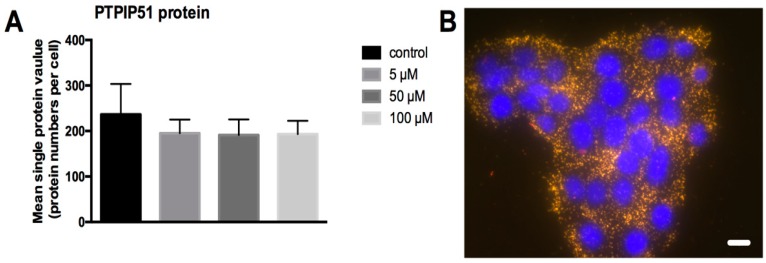
Quantification of total PTPIP51 molecules. (**A**) PTPIP51 protein per cell as determined by Duolink proximity ligation assay (DPLA) using two proximity ligation assay (PLA) probes (plus and minus) both directed against the PTPIP51 antibody in untreated HaCaT cells and cells treated for 24 h with 5 µM, 50 µM and 100 µM LDC-3. No statistical differences could be revealed (*p* > 0.05). Dunnett’s multiple comparisons test was applied; (**B**) DPLA of PTPIP51 in untreated HaCaT cells. Each dot corresponds to a PTPIP51 molecule. Scale bar: 20 µm.

The amount of PTPIP51 was determined by in situ Duolink proximity ligation assay in untreated and 5 µM, 50 µM and 100 µM LDC-3 treated (24 h) HaCaT cells. The test was performed by using the two DPLA probes raised against rabbit immunoglobulins labeled with either the plus or minus oligonucleotides simultaneously. Both DPLA probes recognize the same bound anti-PTPIP51 antibody. By the ligation of the two oligonucleotides and subsequent amplification, a signal is generated proportional to the amount of PTPIP51 protein. As seen in Figure 4A, the number of PTPIP51 molecules remained unchanged despite increasing LDC-3 concentrations (Figure 4A). The right panel gives an example of the DPLA test for control cells. Each yellow dot corresponds to a signal which marks one PTPIP51 protein.

**Figure 5:**
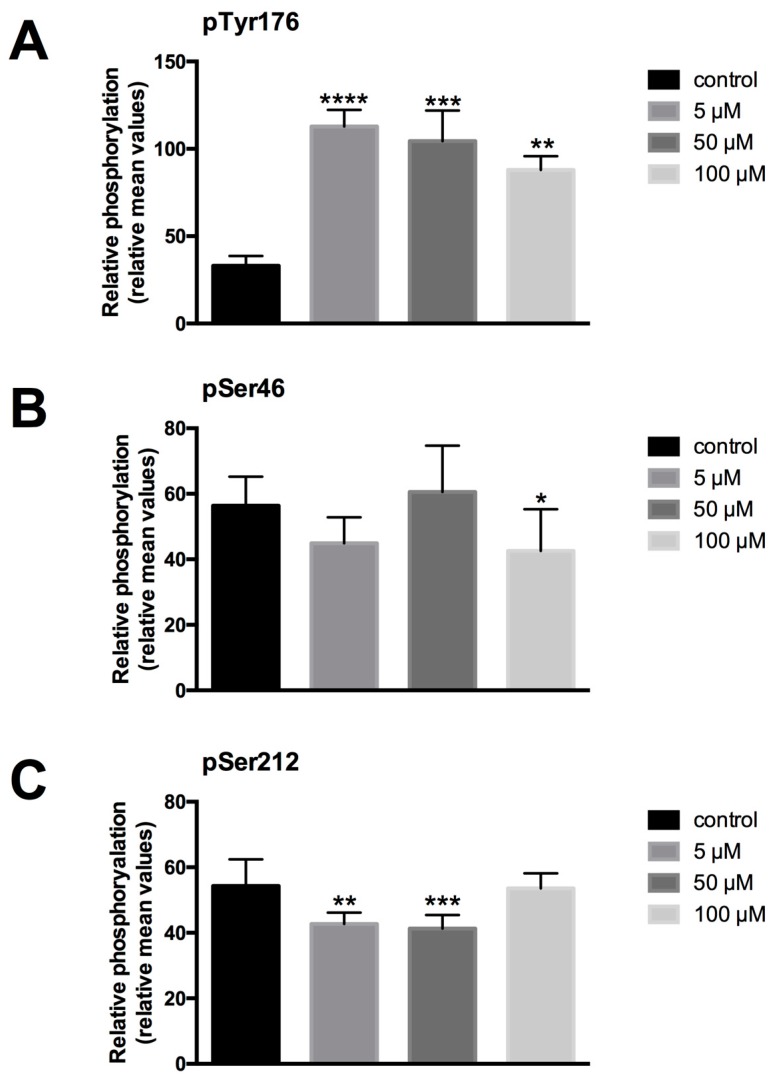
Semiquantitative expression of phospho-Tyr176-PTPIP51 (**A**), phospho-Ser46-PTPIP51 (**B**) and phospho-Ser212-PTPIP51 (**C**) in untreated HaCaT cells and in cells treated for 12 h with 5 µM, 50 µM and 100 µM LDC-3 (*n* = 3). The reaction intensities were recorded by ImageJ [22,23] and the resulting data were analyzed by GraphPad Prism 6 software (GraphPad Software, La Jolla, CA, USA). The significance of results were tested by Dunnett’s multiple comparisons test. * *p* < 0.05, ** *p* < 0.01, *** *p* < 0.001, **** *p* < 0.0001.

To study the impact of LDC-3 treatment on the phosphorylation status of PTPIP51, the Tyr176, Ser46 and Ser212 phosphorylated forms of PTPIP51 were determined by semi-quantitative analyses of immunostainings with antibodies specific to phospho-Tyr176-PTPIP51, phospho-Ser46-PTPIP51 and phospho-Ser212-PTPIP51, respectively. Treating HaCaT cells with LDC-3 at concentrations of 5 µM, 50 µM and 100 µM for 12 h profoundly enhanced the PTPIP51 Tyr176 phosphorylation status to significantly higher values compared to controls (5 µM: *p* < 0.0001; 50 µM: *p* < 0.001) (Figure 5A). A comparable tendency could be revealed by immunoblotting experiments. Yet, 10 µM and 25 µM of LDC-3 decreased the phosphorylation of the Tyr176 residue of PTPIP51 (Supplementary Materials Figure S1).

Under the same conditions, however, no significant changes in the Ser46 phosphorylation status of PTPIP51 was observed (Figure 5B). Interestingly, LDC-3 modulated the phosphorylation status of PTPIP51 Ser212 residue. Applying concentration of 5 µM and 50 µM resulted in a decreased Ser212 phosphorylation, whereas 100 µM left the Ser212 unattended (Figure 5C). Immunoblots investigating the Ser212 phosphorylation status revealed a comparable tendency as observed in the semi-quantitative attempt (Supplementary Materials Figure S1).

### 2.5. LDC-3 Effects on the Time Dependent Interaction Profile of PTPIP51 and PTP1B

Based on the aforementioned high tyrosine phosphorylation status of PTPIP51, we investigated the influence of LDC-3 on the cognate phosphatase of PTPIP51, namely PTP1B, which directly interferes with the complex formation of PTPIP51. The experiments encompassed a time and dose dependent approach. Setting a general comparability for the various interactions of PTPIP51 the values displayed in the line chart were normalized to the corresponding value of the control group (=100%) of each time point for each applied concentration.

**Figure 6:**
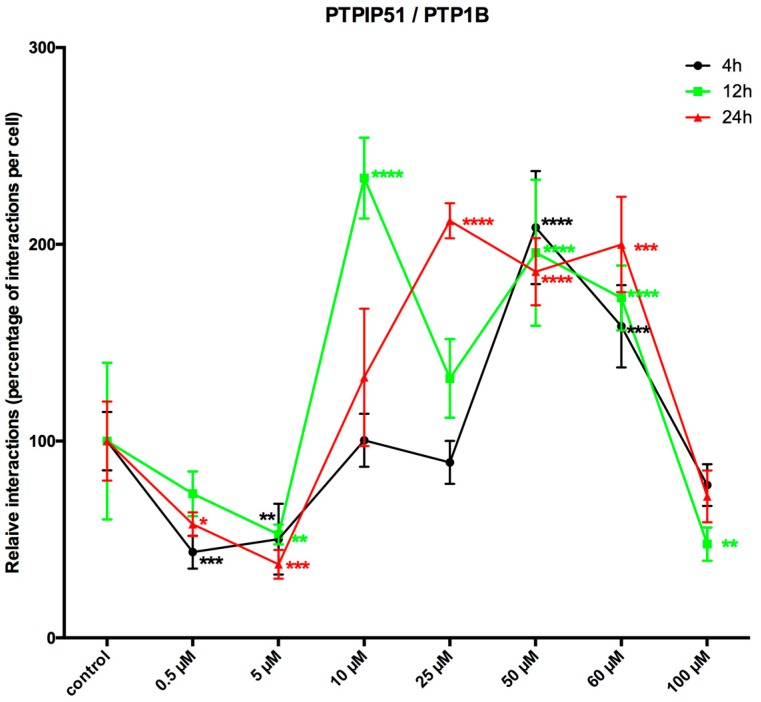
LDC-3 effects on the time dependent interaction profile of PTPIP51 and PTP1B. Quantitative analysis of the Duolink proximity ligation assay of PTPIP51 and PTP1B (*n* = 4). The interactions were evaluated by Duolink Image Tool software in untreated controls and in cells treated with 0.5 µM, 5 µM, 10 µM, 25 µM, 50 µM, 60 µM, 100 µM LDC-3, for 4 h, 12 h and 24 h HaCaT cells. The resulting data were analyzed by GraphPad Prism 6 software (GraphPad Software), significance of results tested by Dunnett’s multiple comparisons test. * *p* < 0.05, ** *p* < 0.01, *** *p* < 0.001, **** *p* < 0.0001.

Compared to controls, the PTPIP51/PTP1B interactions were significantly reduced by the application of low LDC-3 concentrations and short incubation time (4 h) (0.5 µM: *p* < 0.001; 5 µM: *p* < 0.0001), whereas 50 µM and 60 µM significantly increased the number of interactions to supra-normal levels (50 µM: *p* < 0.0001; 60 µM: *p* < 0.001). Yet, the application of 100 µM had no effect on the number of interactions (*p* > 0.05) (Figure 6). If the incubation time was prolonged up to 12 h and 24 h the number of interactions for low LDC-3 concentrations (5 µM and 10 µM) was significantly lower than the values observed in controls (5 µM: *p* < 0.001; 10 µM: *p* < 0.0001) (Figure 6). Concentrations higher than 25 µM led to supranormal PTPIP51/PTP1B interaction levels (50 µM: *p* < 0.001; 60 µM: *p* < 0.05). Yet, the use of 100 µM for 12 h was followed by a sharp decrease in the number of interactions.

Incubating the cells for 24 h with LDC-3 displayed a significant decrease in the PTPIP51/PTP1B complex formation for 0.5 µM and 5 µM LDC-3 (0.5 µM: *p* < 0.05; 5 µM: *p* < 0.001) and a highly significant increase of interactions at concentrations from 25 µM to 60 µM being at 25 µM (*p* < 0.0001), at 50 µM (*p* < 0.001) and at 60 µM (*p* < 0.0001) (Figure 6).

The effects of LDC-4 and LDC-9 on PTPIP51/PTP1B interactions are discussed in the supplementary information (Supplementary Materials Figure S2).

### 2.6. LDC-3 Treatment Induces Altered Protein–Protein Interactions of the Endogenous Interactome of PTPIP51

**Figure 7:**
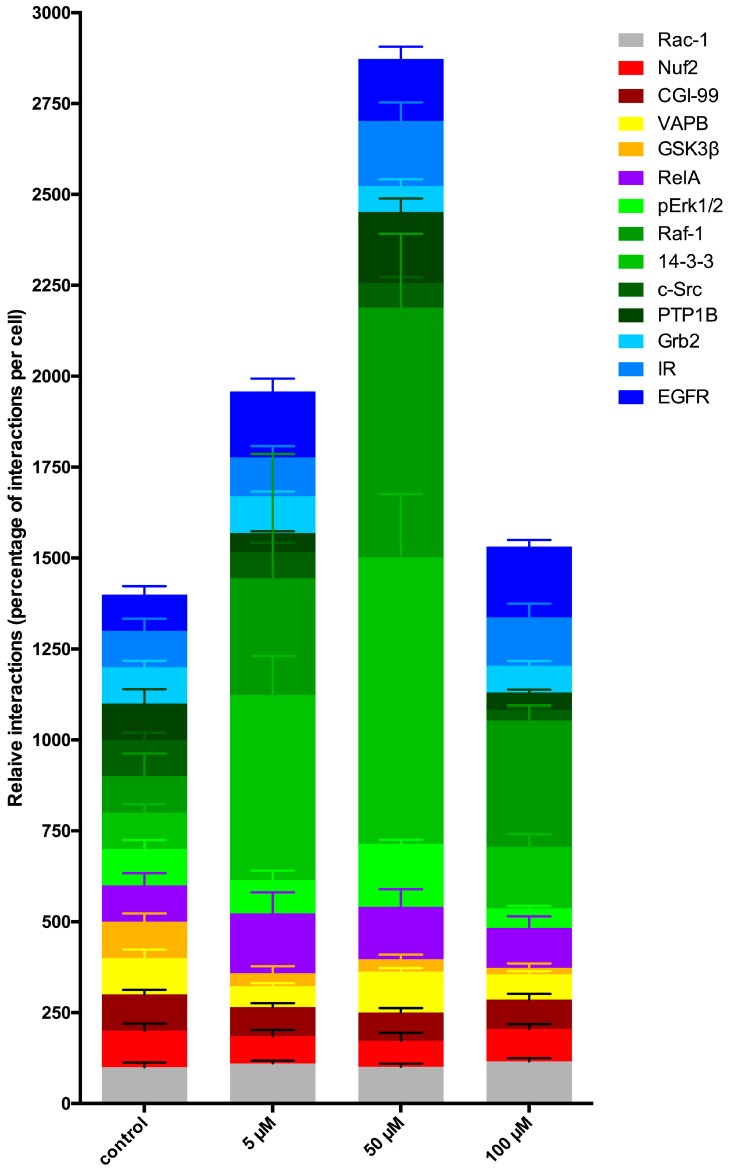
Cellular protein complex shift of PTPIP51 within 14 protein–protein interactions. Quantitative analysis of the Duolink proximity ligation assay of PTPIP51 with 13 different interaction partners in untreated and LDC-3 treated (5 µM, 50 µM and 100 µM) HaCaT cells (*n* = 4). The control values of 14 different PTPIP51 protein interactions were equalized to 100% (first stacked column) and the following columns show the standardized interaction values related to the controls (equaling 100%) for the applied LDC-3 concentrations. LDC-3 annuls the known regulatory phosphorylation mechanism of PTPIP51 with significant impact on the assembly of the PTPIP51 associated protein complexes. LDC-3 forces a protein–protein interaction shift of PTPIP51 and stabilizes the protein within MAPK complex on Raf-1 level through the scaffold protein 14-3-3. The remaining members of the PTPIP51 interactome are less affected by the administration of LDC-3. The color code for the single interactions is given at the upper right. The proteins belonging to the same signaling protein complex are coded by graded shades of the same color: blue corresponds to receptor tyrosine kinases and adapter molecules, green corresponds to the MAPK pathway molecules, red corresponds to mitosis associated proteins. For detailed information to the single interaction and their statistics see Figure 6 and supplementary Figures S3 and S4.

The previous results displaying an increased PTPIP51/PTP1B interaction level are contradicting to the high Tyr176 phosphorylation status of PTPIP51. To explain these findings, the known interactome of PTPIP51 was quantitatively assayed by the Duolink proximity ligation assay. The acquired control values for each single interaction were set to 100% and the number of the corresponding interaction of LDC-3 treated cells were related to the 100% control value which gives the percental quotient of the drug treated cells compared to the untreated control group. In Figure 7 the left column represents the controls of 13 different PTPIP51 protein interactions equalized to 100% and the 3 right columns give the standardized interaction values related to the controls for 5 µM, 50 µM and 100 µM LDC-3 treated cells. This diagram was chosen to illustrate the altered protein–protein interactions of PTPIP51 with its endogenous interactome (Figure 7). An intermediate incubation time of 12 h was selected to cover the main effects of LCD-3 on PTPIP51 protein–protein interactions.

Interestingly, LDC-3 treatment forced PTPIP51 to augment its interactions with the EGF receptor. This effect was statistically significant for all applied concentrations (5 μM: *p* < 0.01; 50 μM: *p* < 0.05; 100 μM: *p* < 0.001) (Figure 7 and Supplementary Materials Figure S3A). Moreover, the PTPIP51/IR interaction values rose at a concentration of 50 μM LDC-3, whereas the other concentrations had no effect on PTPIP51/IR complex formation (Figure 7 and Supplementary Materials Figure S3B). Grb2, the adapter molecule of the two receptor tyrosine kinases, is also part of the PTPIP51 interaction complex. Its PTPIP51 interaction levels were reduced by concentrations of 50 μM and 100 μM LDC-3 (Figure 7 and Supplementary Materials Figure S3D). Beside these two major receptor tyrosine kinases and their adaptor molecule, the complex formation of PTPIP51 with c-Src showed a reduction exclusively at 100 μM LDC-3 (Figure 7 and Supplementary Materials Figure S3C).

These observations go along with a high phosphorylation status of PTPIP51. Thus, the quantitative analysis of the MAPK associated interactions was indispensable.

Treating cells with rising concentrations (5 μM, 50 μM, 100 μM) of LDC-3 for 12 h increased the protein complex formation of PTPIP51 and 14-3-3 in a highly significant manner in comparison to untreated controls up to a concentration of 50 μM (5 μM: *p* < 0.0001; 50 μM: *p* < 0.0001) (Figure 7 and Supplementary Materials Figure S3E). The highest count of interactions was seen for cells treated with 50 μM of LDC-3, whereas application of 100 μM reduced the number of interactions to values near normal, but still significantly higher than the control value (*p* < 0.05) (Figure 7 and Supplementary Materials Figure S3E). A comparable altered interaction profile was observed for the PTPIP51/Raf-1 complex. Values seen for cells treated with 50 μM and 100 μM LDC-3, respectively, were significantly higher than the control values (50 μM: *p* < 0.0001; 100 μM: *p* < 0.05) (Figure 7 and Supplementary Materials Figure S3F). Yet, application of 5 μM had no effect on the PTPIP51/Raf-1 complex formation (Figure 7 and Supplementary Materials Figure S3F). Despite this lacking effect at the lowest LDC-3 concentration, there is a remarkable trend towards a higher interaction level of PTPIP51/Raf-1, although not statistically significant (Figure 7 and Supplementary Materials Figure S3F).

The interactions of 14-3-3/Raf-1 corroborated the reaction pattern seen for PTPIP51/Raf-1 complex formation. Highly significant differences to normal values were seen for the 14-3-3/Raf-1 interactions only at 50 μM LDC-3 (*p* < 0.0001) (Supplementary Materials Figure S3H).

An analogous pattern was displayed for the interaction of PTPIP51 and pErk1/2 in LDC-3 treated cells. Application of 5 μM LDC-3 did not alter the number of interactions compared to the controls, whereas 50 μM resulted in a significantly enhanced number of interactions (*p* < 0.05). The number of interactions was reduced far below the values seen in untreated controls by using 100 μM LDC-3 (*p* < 0.05) (Figure 7 and Supplementary Materials Figure S3G).

The interaction profiles with two proteins of the mitotic process—CGI-99 and Nuf2—were negatively related to the rising concentrations of LDC-3 applied for 12 h. The interaction PTPIP51/CGI-99 highly significantly differed from the control values (5 μM: *p* < 0.05; 50 μM: *p* < 0.01; 100 μM: *p* < 0.5) (Figure 7 and Supplementary Materials Figure S4A). The complex formation of PTPIP51 with Nuf2, the kinetochore anchoring protein, also showed a reduction for 5 μM (*p* < 0.05) and 50 μM (*p* < 0.01), but not for 100 μM (*p* > 0.05) (Figure 7 and Supplementary Materials Figure S4B).

The PTPIP51 interaction partners of the NFkB signaling, of the mitochondria and ER-calcium homeostasis as well as the cell motility signaling pathways also showed slight changes in complex formation (Figure 7 and Supplementary Materials Figure S4). LDC-3 led to a transient significant increase (*p* < 0.05) in the number of interactions of PTPIP51 and the RelA subunit of NFκB at a concentration of 5 μM LDC-3. A further augmentation to 50 μM and 100 μM LDC-3 reversed the pattern to more normal values (*p* > 0.05) (Figure 7 and Supplementary Materials Figure S4F). Treatment of HaCaT cells with 5 μM LDC-3 for 12 h significantly reduced the interactions of PTPIP51 with VAPB by almost 50% (5 μM: *p* < 0.01) compared to controls. Application of a concentration of 50 μM LDC-3 restored the interaction profile to approximately normal values, whereas a concentration of 100 μM significantly (*p* < 0.05) decreased the VAPB interaction with PTPIP51 (Figure 7 and Supplementary Materials Figure S4C). In addition, PTPIP51 interacts with the GSK3β in the HaCaT cell line. For all tested concentrations of LDC-3 the interaction of PTPIP51 and GSK3β was significantly reduced compared to the control group (5 μM and 50 µM: *p* < 0.01; 100 µM: *p* < 0.001) (Figure 7 and Supplementary Materials Fig S4D).

HaCaT cells treated with 100 μM LDC-3 for 12 h displayed a highly significant augmentation (*p* < 0.01) in the interaction of PTPIP51 and Rac1 (Figure 7 and Supplementary Materials Figure S4E).

## 3. Discussion

To date, PTPIP51 is a relatively well described protein regarding its multiple participations within cellular signaling pathways. In the current study, we give an overview of physiological protein complex clustering in HaCat cells. A number of these signaling pathways are inflicted in pathogenic processes. Such interaction profiles build the bases for targeted therapy of deteriorated signal transduction. Yet, up until now it was impossible to directly target PTPIP51 protein function and, therefore, the associated interactome. In a high-throughput screen for small molecules interfering with the Hedgehog signaling pathway, the aminothiazole LDC-3 was identified to bind to PTPIP51 [21,24]. The specificity of the LDC-3 binding to PTPIP51 was corroborated in a yeast-three-hybrid assay [21]. Furthermore, in own functional experiments to better understanding the efficacy and molecular mechanism of LDC-3 effects, we employed a knock down approach. These knock-down experiments of PTPIP51 abolished the effect of LDC-3 on the MAPK pathway. Therefore, we claim that LDC-3 is able to modulate PTPIP51 protein interactions.

Physiological regulation of PTPIP51 protein–protein interactions is mediated by the tyrosine and serine phosphorylation status of PTPIP51 [4,9]. If phosphorylated at its Tyr176 residue PTPIP51 interaction with Raf-1 through 14-3-3 is inhibited and, therefore, PTPIP51 is incapacitated to stimulate the MAPK pathway [4,9]. This inhibition is mediated by receptor tyrosine kinases, e.g., the EGFR [4]. Ser212 phosphorylation antagonizes the aforementioned effect by augmenting the affinity of PTPIP51 to Raf-1 [9].

LDC-3 treatment of HaCaT cells strongly enhanced the MAPK binding affinity of PTPIP51 in a dose dependent manner regardless of its regulatory phosphorylation status. PTPIP51 showed a high phosphorylation level at Tyr176 in HaCaT cells, paralleled by the enhanced interaction of PTPIP51/EGFR and PTPIP51/IR, respectively. The detected decrease of the Tyr176 phosphorylation by immunoblotting compared to the semi-quantitative analyses may due to the incomplete registration of the various isoforms of PTPIP51. Facing the high Tyr176 phosphorylation status, the PTPIP51/PTP1B protein complex is augmented. This regulatory circuit is disabled by LDC-3. Therefore, LDC-3 forces PTPIP51 into the MAPK protein cluster and stabilizes the interactions of PTPIP51 with 14-3-3 and Raf-1, respectively. Consequently, the MAPK pathway activity is augmented as verified by immunoblotting experiments. Interestingly, sustained p42/p44-MAPK (Erk1/2) activation can lead to anti-proliferative effects with subsequent induction of apoptosis [25]. LDC-3 also prohibited the mitotic function of PTPIP51 by reducing CGI-99 and Nuf2 interaction, although PTPIP51 shows high Tyr176 phosphorylation levels normally seen during mitosis [3].

The MAPK pathway regulates cellular proliferation, differentiation, apoptosis and migration. These processes play a central role in human diseases, not only in tumorigenesis, but also in neurodegeneration, making it a main target for therapeutic intervention [26,27].

Recent studies linked the interaction of PTPIP51/VAPB to the GSK3β activation status in a neurodegenerative disease, namely ALS [28]. If GSK3β is activated it perturbs the PTPIP51/VAPB interaction and therefore the attachment of mitochondria to the mitochondria associated membrane (MAM) of the ER. This mechanism leads to an abnormal calcium homeostasis and might be involved in the induction of apoptosis [28]. In the current study, we verified a direct interaction of PTPIP51 with GSK3β. LDC-3 also perturbs the mitochondria/ER binding by preventing PTPIP51/VAPB complex formation. The reduced PTPIP51/GSK3β interactions presumably maintain cell survival by the protection of the residual PTPIP51/VAPB complexes followed by the inactivation of GSK3β. LDC-3 treatment also reduced the mitochondrial metabolic rate as an additional expression for the perturbed interaction of PTPIP51 with VAPB. Interestingly, Akt can deactivate GSK3β by phosphorylating its Ser9 residue [29]. Yet, the activation status of Akt is depleted under the influence of LDC-3. This effect can be observed for concentrations of 5 µM and higher. The lowest concentration of 0.5 µM LDC-3 has no effect on the Akt activation status, whereas p42/p44-MAPK (Erk1/2) is activated. We assumed that the MAPK signaling cascade is the preferentially modulated pathway by the LDC-3 targeted PTPIP51 in µmolar concentrations. Yet, the exact mechanism remains to be solved. PKCα was further analyzed in regard to its activation status. PKCα directly targets the inhibitory Ser9 residue by phosphorylating it [30]. PKC can deactivate Akt in murine keratinocytes [31] and was probably activated by the altered calcium release due to the disturbed interaction of PTPIP51/VAPB interaction. This may be followed by a reduction of the MAM area. Notably, PKCα itself interacts with PTPIP51 [4]. In conclusion, we assume that the PTPIP51/VAPB complex formation is monitored by the activity of PKCα and GSK3β.

A probable mechanism explaining the described altered interaction of PTPIP51 with its endogenous interactome might be an allosteric switch within the PTPIP51 protein structure. PTPIP51 exerts its function by specific binding sites [2,32]. These binding sites can either be exposed or concealed by LDC-3, thus facilitating or impairing interactions.

Protein complexes and their underlying protein–protein interactions play critical roles in normal cellular function and malignant transformation [33]. For two reasons, detailed insights into variations and alteration in protein complexes are strongly needed: (1) If cellular protein complexes are not sufficiently characterized, normal cellular function and probably malignant transformations will not be fully understood. (2) Lately, small molecule drugs affecting protein–protein interactions were developed for anti-cancer therapy [34]. In this context, it is very critical to identify and analyze altered protein complex formation to determine the mode of action as well as the effectiveness of the new drug. In further studies, it will be crucial to examine how the strong stabilization of the PTPIP51/14-3-3/Raf1 protein complex by LDC-3 impacts a malignantly transformed cellular system with deregulated signal transduction in regard to the MAPK pathway. A study analyzing the effects of LDC-3 on breast cancer cells has already been performed proving a possible therapeutical application of LDC-3 [35].

Moreover, LDC-3 or a pharmacological derivative of it might also be a therapeutic option for the treatment of ALS normalizing the calcium homeostasis.

The intervention by LDC-3 in the PTPIP51 interactome and the associated cross-talks offers an enormous potential to interfere in deranged signal transduction pathways and may, therefore, give many options for therapeutic applications.

## 4. Materials and Methods

### 4.1. Compounds

LDC-3, LDC-4 and LDC-9 were synthesized by the Lead Discovery Center, GmbH, Dortmund, Germany; the chemical structures and broad specificity experiments have been submitted for publication. Stock solutions, dissolved in DMSO, were stored at −80 °C. For treatment, the substances were diluted in culture media and applied in the indicated concentrations.

### 4.2. Cell Culture

All experiments of this study were performed with HaCaT cells kindly provided by Dr. Teschemacher (Department of Pharmacology, Justus-Liebig-University, Giessen, Germany) with the permission of Dr. Fusenig (DKFZ, Heidelberg, Germany, MTA number L-4598). Cells were kept at 37 °C in a humidified 5% CO_2_ atmosphere and were cultured in Roswell Park Memorial Institute 1640 (RPMI1640) medium (PAA, Paching, Austria) supplemented with 10% fetal calf serum (FCS), 1% penicillin and 1% streptomycin (ThermoFisher Scientific, Langenselbold, Germany). For experiments cells were grown on culture slides coated with FCS until near confluency. Subsequently, the medium was removed and the cells were treated with LDC-3 containing RMPI1640. The control cells were solely incubated in RPMI1640 medium. The reactions were terminated by withdrawing the medium, adding ice cold phosphate buffered saline (PBS, pH 7.4) and fixation of the cells with 4% paraformaldehyde. Cells were subsequently fixed with methanol and immunocytochemistry was applied.

### 4.3. Determination of Mitochondrial Metabolic Rate by the MTT Assay

Mitochondrial metabolic rate was tested using an MTT assay (Roche Diagnostics, Mannheim, Germany). The assay was performed according to the manufacture’s manual. In short: HaCaT cells (10,000 cells/well) were cultured in flat-bottomed 96-well tissue culture plates either with medium (controls) or with LDC-3 supplemented medium. After preincubation of 24 h the MTT labeling solution was added and incubated for further 6 h. The reaction was stopped by adding the solubilizing solution overnight. The assay was analyzed by reading absorbance at 560 nm.

### 4.4. BrdU Proliferation Assay

The BrdU proliferation assay was performed according to the manufacture’s manual (Cell signaling technology, Frankfurt, Germany). In short: HaCaT cells (10,000 cells/well) were cultured in flat-bottomed 96-well tissue culture plates either with medium (controls) or with LDC-3 supplemented medium. After preincubation of 24 h without or with the LDC effectors BrdU was added and incubated for further 24 h. The reaction was stopped by fixation and lysis of the cells followed by the addition of a horseradish peroxidase (HRP)-linked antibody and tetramethylbenzidine (TMB) substrate. Proliferation was analyzed by reading absorbance at 450 nm.

### 4.5. Immunoblotting

Samples of HaCat cell lysates (*n* = 3) were separated on Mini-PROTEAN TGX Stain-Free Precast Gels (Bio-Rad, München, Germany). Transfer on an Immobilon P membrane (Millipore, Billerica, MA, USA) was performed according to manufacturer’s instructions using the Bio-Rad Trans-Blot Turbo Transfer System (Bio-Rad) with the settings for mixed molecular weight proteins. The membrane was blocked with 1× Rotiblock for 1 h at room temperature. Incubation with polyclonal rabbit anti-PTPIP51, anti-pMAPK or anti-CDK-1 was done overnight at 4 °C. HRP-conjugated anti-rabbit immunoglobulins were applied for 1 h at room temperature diluted in 1× Rotiblock. The reaction was visualized with the enhanced chemiluminescence (ECL) prime substrate. For documentation the Bio-Rad ChemiDoc Touch Imaging System (Bio-Rad) was used. Calibration was performed with a molecular weight marker suitable for chemiluminescence (Life technologies GmbH, Darmstadt, Germany). The blots were equalized to the obtained stain-free blot for comparison using the Bio-Rad Image Lab (Bio-Rad). Therefore, no loading control is required. The stain-free blots and the quantification data for the shown experiments are given in the supplementary information.

### 4.6. siRNA Experiment

siRNA constructs were obtained from Origene (Rockville, MD, USA). Cells were grown in flat-bottomed 24-well tissue culture plates for 24 h with a starting cell number of 100,000 cells per well before transfection. The provided siRNA constructs were initially dissolved in the supplied siRNA dilution buffer to a final concentration of 20 µM. For RNA interference (RNAi) experiments the constructs were further diluted using Opti-Mem and the adequate amount of siTrans 1.0 transfection reagent according to the manufacturer’s protocol resulting in a final working dilution of 10 nM.

HaCat cells (*n* = 3) were incubated for 24 h with one of the siRNA contructs and the scramble control. A subset of the cells was left untreated for another 24 h, whereas the other subgroup was treated with 50 µM of LDC-3 for 24 h. The reaction was terminated by the administration of the NuPAGE LDS Sample Buffer (Thermo Fischer Scientific, Waltham, MA, USA).

### 4.7. Quantification of PTPIP51 Protein

To evaluate the influence of LDC-3 on the amount of PTPIP51 protein per single cell the DPLA test was applied, using two PLA probes (DPLA rb plus/minus) directed against the primary rabbit antibody. Both PLA probes bind to the same antibody and are ligated. The signal is several hundred-fold enhanced by rolling circle amplification. Each interaction is presented by a dot and corresponds to one PTPIP51 molecule.

### 4.8. PTPIP51 Antibody

The PTPIP51 antibody (P51ab) was produced as described previously [8].

### 4.9. Immunohistochemistry

Immunohistochemistry was performed as previously described by Koch et al. [8]. Prior to immunostaining nonspecific binding sites were blocked with 0.1 M phosphate buffered saline (pH 7.4) containing 5% bovine serum albumin and 5% normal goat serum for 1 h. Samples were incubated overnight at room temperature with primary antibodies (Supplementary Materials Table S1) diluted in PBS, followed by washing in PBS (three times for 10 min) and subsequent incubation for 1 h at room temperature with the corresponding secondary antibodies (Supplementary Materials Table S1). The slides were washed in PBS, coverslipped in carbonate buffered glycerol at pH 8.6 and evaluated either by epifluorescence microscopy or by sequential confocal laser scanning microscopy. (Carl Zeiss, Jena, Germany). PTPIP51 (aa131–470) and peptide specific PTPIP51 antibodies (Table S1) were visualized either by an Alexa Fluor 555 coupled anti-rabbit secondary antibody (Table S1) or a Cy3 coupled anti-guinea pig antibody (Table S1). Primary mouse antibodies used for double staining were visualized by using an Alexa Fluor 488 coupled anti-mouse secondary antibody (Table S1). Nuclei were displayed by DAPI.

### 4.10. Semiquantification of Protein Expression

For the semiquantive analysis of the expressed protein, images were taken under standard conditions (time of exposition). The stored images were analyzed by the ImageJ program, measuring the immunfluorescence intensities of immunostained HaCaT cells by encircling the stained cells. The intensities of 800–1000 cells/group were measured and averaged. The resulting mean values of ten images for Tyr176, Ser46 and Ser212 phosphorylated PTPIP51 were referred to as relative units and plotted in a diagram. The experiments were repeated three times (*n* = 3).

### 4.11. Epifluorescence Microscopy

The Axioplan 2 fluorescence microscope equipped with Plan-Apochromat objectives (Carl Zeiss Jena, Germany) was used for photo documentation. For visualization of the secondary antibody Alexa Fluor 555 an excitation filter with a spectrum of 530–560 nm and an emission filter with a spectrum 572–647 nm were used. Alexa Fluor 488 was visualized by an excitation filter with a range of 460–500 nm and an emission filter with a range of 512–542 nm.

### 4.12. Peptide Specific Phospho-Tyr176 PTPIP51 Antibody

For analysis of the tyrosine phosphorylation state of PTPIP51, an antibody (BioLux, Stuttgart, Germany) to the Tyr176 phosphorylated sequence DAESEGGYTTANAE was used (PTPIP51-PTyr176). The identity and the purity of the synthetized antigenic peptide was approved by electrospray ionization mass spectrometry (ESI-MS) and ultraviolet (UV)-analysis. Guinea pigs were immunized with the keyhole limpet hemocyanin (KLH)-conjugated peptide. The specificity of the antibody was tested by enzyme-linked immunosorbent assay (ELISA) and Western blot. To verify the use of the peptide specific phospho-antibody for immunostaining, preabsorption experiments were performed [3].

### 4.13. Peptide Specific Phospho-Serine 46 and 212 PTPIP51 Antibody

For analysis of the serine phosphorylation state of PTPIP51, an antibody (Genosphere Biotechnologies, Paris, France) to the serine 46 phosphorylated sequence CQRHGRSQ[pS]LPNS and an antibody (Genosphere Biotechnologies) to the Ser212 phosphorylated sequence CETVKMGRKD[pS]LDLE were used.

### 4.14. Confocal Laser Scanning Microscopy

Confocal images of HaCaT cells were obtained with a Zeiss confocal laser scanning microscope (CLSM, LSM 800, Carl Zeiss). Confocal images of Cy3 fluorescence were acquired using Plan-Apochromat 40×/1.1 water objective. The laser voltage was set to 550 V for all analyses. The pinhole diameter was set to 1 Airy unit. Acquisition of confocal images was done using the Zeiss Zen 2.1 software. Subsequently the acquired images were processed by Arivis vision4D 2.12 (Arivis, München, Germany) for producing 3D reconstructions.

### 4.15. Duolink Proximity Ligation Assay (DPLA)

In situ interactions were detected by the proximity ligation assay kit Duolink (PLA probe anti-rabbit minus, PLA probe anti-mouse plus (Sigma-Aldrich, St. Louis, MO, USA); Dection Kit Orange, (Sigma-Aldrich)). The DPLA probe anti-rabbit minus binds to the PTPIP51 antibody, whereas the PLA probe anti-mouse plus binds to the antibody against the probable interaction partner (Supplementary Materials Table S1), respectively. The Duolink proximity ligation assay secondary anti-bodies generate only a signal when the two DPLA probes have bound, which only takes place if both proteins are closer than 40 nm, indicating their interaction [36]. Paraformaldehyde-fixed HaCaT cells were pre-incubated with blocking agent for 1 h. After washing in PBS for 10 min, primary PTPIP51 antibody (1:1000) was applied to the samples. Primary antibodies to the interacting partner (Supplementary Materials Table S1) were used for proving the interaction by co-incubation with the PTPIP51 antibody. The specificity of the antibodies is documentated in the supplementary Table S1 by giving the appropriate scientific reference. Incubation was done overnight in a pre-heated humidity chamber. Slides were washed three times in PBS for 10 min. Duolink PLA probes detecting rabbit or mouse antibodies were diluted in the blocking agent in a concentration of 1:5 and applied to the slides followed by incubation for 1 h in a pre-heated humidity chamber at 37°C. Unbound DPLA probes were removed by washing two times in PBS for 5 min. The samples were incubated with the ligation solution consisting of Duolink II Ligation stock (1:5) and Duolink Ligase (1:40) diluted in high purity water for 30 min at 37 °C. After ligation the Duolink Amplification and Detection stock, diluted 1:5 by the addition of polymerase (1:80), was applied to the slides for 100 min. Afterwards the slides were incubated with DAPI for the identification of nuclei. After the final washing steps the slides were dried and coverslips were applied. Quantification was done with the DuoLink Image Tool v1.0.1.2 (OlinkBioscience, Uppsala, Sweden). The signal threshold was adjusted to 100 and the pixel size for spot detection to 5 pixels for each picture.

### 4.16. Statistical Analysis

The quantified DPLA spots were calculated per cell (number of dots/cell) for each picture. The results were standardized to the number of interactions/cell. Approximately 2000 cells were analyzed of four independent experiments for all protein–protein interactions and PTPIP51 protein quantification (*n* = 4). The values were subsequently analyzed by GraphPad Prism 6 (GraphPad Software) using the Dunnett’s multiple comparisons test. Results were considered as significant with *p* < 0.05.

## Supplementary Materials

The following are available online at www.mdpi.com/2218-273X/7/3/55/s1, Figure S1: Immunoblot analyses of the phospho-Tyr176 and phospho-Ser212 status of PTPIP51, Figure S2: MTT assay of HaCaT cells treated with LDC-4 and LDC-9 and the LDC-4 and LDC-9 effects on the time dependent interaction profile of PTPIP51 and PTP1B, Figures S3: Effects of LDC-3 on the receptor tyrosine kinase signaling and associated adapter molecules and on the MAPK signaling pathway, Figures S4: Effects of LDC-3 on the mitotic interactome of PTPIP51, the NFkB pathway, the Ca^2+^ homestasis and the cell motility, Figure S5: 3D reconstruction of HaCaT cells treated with LDC-3 and immunostained with the antibody specific to phospho-Tyr176-PTPIP51, Figure S6: Stain-free blot for quantification, Figure S7: Semiquantitative analyses of the Tyr176 phosphorylation status of PTPIP51, Figure S8: Semiquantitative analyses of the Ser46 phosphorylation status of PTPIP51, Figure S9: Semiquantitative analyses of the Ser212 phosphorylation status of PTPIP51, Table S1: Antibody list.
