# Supplementary material for: Altered Protein Interactions of the Endogenous Interactome of PTPIP51 towards MAPK Signaling
Source: Biomolecules. 2017 Jul 21;7(3):55. doi: 10.3390/biom7030055 (PMC5618236; doi:10.3390/biom7030055)
Supplement: Supplementary file 1 [file biomolecules-07-00055-s001.pdf]

Table S1. Antibody list. List of all antibodies used in this study.

	Immunogen	Antibody Source	Clone	Dilution	Manufacturer	Application	Reference
**PTPIP51 (P51ab)** The specificity was controlled by preabsorption experiments.	Human recombinant PTPIP51 protein encoding amino acids (aa) 131-470	Rabbit polyclonal		1:500	Prof. HW Hofer, Biochemical Department, University Konstanz, Germany	WB, DPLA	[1-4]
**PTPIP51-anti-pTyr176 PTPIP51** The specifity was controlled by RP-HPLC MassSpectrum	KLH-coupled peptide with the sequence: CDAESEGG[pT]TANAE	Guinea pig polyclonal		1:1000	Biolux, Stuttgart, Germany	IHC	[4]
**PTPIP51 anti-pSer46 PTPIP51** The specifity was controlled by RP-HPLC MassSpectrum	KLH-coupled peptide with the sequence: CQRHGRSQ[pS]LPNS	Guinea pig polyclonal		1:500	Genosphere Biotechnologies, Paris, France	IHC	[5]
**PTPIP51 anti-pSer212 PTPIP51**	KLH-coupled peptide with the sequence:	Rabbit polyclonal		1:500	Genosphere Biotechnologies, Paris, France	IHC	

The specifity was controlled by RP-HPLC MassSpectrum	CETVKMGRKD[pS] LDLE						
**PTP1B**	Recombinant protein corresponding to aa 1-321 of human PTP1B	Mouse monoclonal	107AT5 31	1:200	Abnova<br>Cat.# MAB1152<br>Lot: SDG070208D	DPLA	[6]
**14-3-3ß**	Epitope mapping between aa 220-244 at the C-terminus of human 14-3-3ß	Mouse monoclonal	A-6	1:100	Santa Cruz Biotechnology<br>Cat.# sc-25276<br>Lot: F1011	DPLA	[7]
**Raf-1**	Epitope mapping the C-terminus of Raf-1	Mouse monoclonal	E-10	1:100	Santa Cruz Biotechnology<br>Cat.# sc-7267<br>Lot: E0411	DPLA	[8]
**EGFR**	raised against plasma membranes of A431 cells	Mouse monoclonal	2E9	1:100	Santa Cruz Biotechnology<br>Cat.# sc-57091<br>Lot: B1307	DPLA	[9]
**ERK1/2**	raised against synthetic peptide corresponding to	Mouse Monoclonal	MK12	1:100		DPLA	[10]

**(p42/44)**	aa336-356 of human ERK1				Merck Millipore<br>Cat.# 05-1152<br>Lot: 1956740		
**pERK**	Epitope corresponding to a sequence containing Tyr204 phophorylated ERK of human origin	Mouse monoclonal	E-4	1:100	Santa Cruz Biotechnology<br>Cat. # sc-7383<br>Lot: F2613	DPLA	[11]
**CGI99**	epitope mapping at the N-terminus of CGI-99 of human origin	Goat polyclonal	N-14	1:100	Santa Cruz Biotechnology<br>Cat. # sc-104834<br>Lot: C0310	DPLA	[4]
**Nuf-2 (cdcA1)**	raised against aa 1–300 mapping at the N-terminus of CdcA1 human origin	Mouse monoclonal	E-6	1:100	Santa Cruz Biotechnology<br>Cat.# sc-271251<br>Lot: E2510	DPLA	[12]
**RelA**<br>validated in EMSA, FC, ICC, IF, IHC, IHC(P), WB to detect NFκB	Specfic for an epitope overlapping the nuclear location signal of the p65 subunit of the NFKB heterodimer	Mouse monoclonal	12H11	1:100	Millipore<br>Cat.# MAB3026<br>Lot: 2580674	DPLA	[13]

also known as Rel A							
**Rac1**<br><br>routinely evaluated by immunoblot on rat brain microsomal protein validated for use in Immunohistochemistry (IHC), Immunoprecipitation (IP) and Western Blotting (WB) for the detection of Rac1 protein	Recombinant protein containing the full length human Rac1	Mouse monoclonal	23A8	1:100	Merck-Millipore<br><br>Cat.# 05-389<br><br>Lot: E0411	DPLA	[14]
**VAPB**	E. coli-derived recombinant human<br><br>VAP-B. Ala2-Pro132	Mouse monoclonal	736904	1:100	R&D systems<br><br>Cat.# MAB58551	DPLA	[15]
**IR (ß-subunit)**<br><br>Routinely evaluated by Western Blot on NIH/3T3 lysates.	Recombinant-fragment including C-terminal 100 aa of human insulin receptor ß-subunit	Mouse monoclonal	CT-3	1:100	Merck-Millipore Cat.#05-1104	DPLA	[16]

Western Blot Analysis: 1:500 dilution of this lot detected Insulin Receptor, beta subunit on 10 µg of NIH/3T3 lysates.							
**Grb2**	raised against amino acids 54-164 mapping to a central domain of GRB2 of mouse origin	Mouse monoclonal	C-7	1:100	Santa Cruz Biotechnology<br>Cat.# sc-8034<br>Lot: 2614	DPLA	[17]
**c-Src**	raised against full-length recombinant c-Src of human origin	Mouse monoclonal	H-12	1:100	Santa Cruz Biotechnology<br>Cat.# sc-5266<br>Lot: K2509	DPLA	[18]
**GSK3β**	raised against amino acods 345-420 mapping at the C-terminus of GSK3β of human origin	Mouse monoclonal	E11	1:100	Sanat Cruz Biotechnology<br>Cat.# sc-377213<br>Lot: A3114	DPLA	[19]
**Phospho-Akt (Ser473)**	a synthetic phosphopeptide corresponding to	Rabbit monoclonal	D9E	1:2500	Cell signaling technology	WB	[20]

	residues surrounding Ser473 of mouse Akt				Cat.# 4060<br>Lot: 19		
**Phospho-p42/p44 MAPK**	a synthetic phosphopeptide corresponding to residues surrounding Thr202/Tyr204 of human p44 MAP kinase	Rabbit monoclonal	D13.14.4E	1:2500	Cell signaling technology<br>Cat.# 4370<br>Lot: 12	WB	[21]
**Phospho-cdc2 (Tyr15) (CDK1)**	synthetic phosphopeptide corresponding to residues surrounding Tyr15 of human cdc2	Rabbit monoclonal	10A11	1:2500	Cell signaling technology<br>Cat.# 4539<br>Lot:2	WB	[22]
**Phospho-PKCα (Thr638)**	A synthetic phospho-peptide corresponding to residues surrounding Thr638 of human PKC alpha.	Rabbit monoclonal	E195	1:2500	Abcam<br>Cat.# ab32502<br>Lot: GR13679-1	WB	[23]

**Phospho-GSK3β (S9)**	synthetic phosphopeptide corresponding to the sequence of human GSK-3β.	Rabbit monoclonal	5B3	1:2500	Cell signalling technology<br>Cat.# 9323<br>Lot: 13	WB	[24]
**Alexa 555 Coupled to anti-rabbit antibody**	Rabbit gamma immunoglobins heavy and light chains	Goat polyclonal		1:1600	Life Technology<br>Cat.# A21428<br>Lot:	IHC	
**Alexa 488 Coupled to anti-mouse antibody**	IgG heavy chains from mouse	Goat polyclonal		1:800	Life Technology<br>Cat# A11029<br>Lot:	IHC	
**Cy3 donkey anti-guinea pig**	IgG (H+L)<br>from guinea pig	Donkey<br>polyclonal		1:400	Dianova<br>Cat.# 706166148<br>Lot:	IHC	
**HRP goat anti-rabbit**	IgG isolated from rabbit serum	Goat polyclonal		1:5000	Dako<br>Cat.# P0448<br>Lot: 20027913	WB	

References:

1. Barop, J.; Barop, J.; Sauer, H.; Sauer, H.; Steger, K.; Steger, K.; Wimmer, M.; Wimmer, M. Differentiation-dependent PTPIP51 expression in human skeletal muscle cell culture. *J Histochem Cytochem* **2009**, *57*, 425–435.
2. Stenzinger, A.; Kajosch, T.; Tag, C.; Porsche, A.; Welte, I.; Hofer, H.-W.; Steger, K.; Wimmer, M. The novel protein PTPIP51 exhibits tissue- and cell-specific expression. *Histochem Cell Biol* **2005**, *123*, 19–28.
3. Koch, P.; Viard, M.; Stenzinger, A.; Brobeil, A.; Tag, C.; Steger, K.; Wimmer, M. Expression profile of PTPIP51 in mouse brain. *J. Comp. Neurol.* **2009**, *517*, 892–905.
4. Brobeil, A.; Graf, M.; Eiber, M.; Wimmer, M. Interaction of PTPIP51 with Tubulin, CGI-99 and Nuf2 During Cell Cycle Progression. *Biomolecules* **2012**, *2*, 122–142.
5. Brobeil, A.; Bobrich, M.; Tag, C.; Wimmer, M. PTPIP51 in protein interactions: regulation and in situ interacting partners. *Cell Biochem. Biophys.* **2012**, *63*, 211–222.
6. Brobeil, A.; Graf, M.; Oeschger, S.; Steger, K.; Wimmer, M. PTPIP51-a myeloid lineage specific protein interacts with PTP1B in neutrophil granulocytes. *Blood Cells Mol. Dis.* **2010**, *45*, 159–168.
7. Yam, P. T.; Kent, C. B.; Morin, S.; Farmer, W. T.; Alchini, R.; Lepelletier, L.; Colman, D. R.; Tessier-Lavigne, M.; Fournier, A. E.; Charron, F. 14-3-3 proteins regulate a cell-intrinsic switch from sonic hedgehog-mediated commissural axon attraction to repulsion after midline crossing. *Neuron* **2012**, *76*, 735–749.
8. Bunda, S.; Burrell, K.; Heir, P.; Zeng, L.; Alamsahebpour, A.; Kano, Y.; Raught, B.; Zhang, Z.-Y.; Zadeh, G.; Ohh, M. Inhibition of SHP2-mediated dephosphorylation of Ras suppresses oncogenesis. *Nat Commun* **2015**, *6*, 8859.
9. Downward, J.; Parker, P.; Waterfield, M. D. Autophosphorylation sites on the epidermal growth factor receptor. *Nature* **1984**, *311*, 483–485.
10. Chen, K.-T.; Tsai, M.-H.; Wu, C.-H.; Jou, M.-J.; Wei, I.-H.; Huang, C.-C. AMPA Receptor-mTOR Activation is Required for the Antidepressant-Like Effects of Sarcosine during the Forced Swim Test in Rats: Insertion of AMPA Receptor may Play a Role. *Front Behav Neurosci* **2015**, *9*, 162.
11. Giri, K.; Pabelick, C. M.; Mukherjee, P.; Prakash, Y. S. Hepatoma derived growth factor (HDGF) dynamics in ovarian cancer cells. *Apoptosis* **2016**, *21*, 329–339.
12. Sugimasa, H.; Taniue, K.; Kurimoto, A.; Takeda, Y.; Kawasaki, Y.; Akiyama, T. Heterogeneous nuclear ribonucleoprotein K upregulates the kinetochore complex component NUF2 and promotes the tumorigenicity of colon cancer cells. *Biochem. Biophys. Res. Commun.* **2015**, *459*, 29–35.
13. Díaz, L.; Martínez-Bonet, M.; Sánchez, J.; Fernández-Pineda, A.; Jiménez, J. L.; Muñoz, E.; Moreno, S.; Álvarez, S.; Muñoz-Fernández, M. Á. Bryostatin activates HIV-1 latent expression in human astrocytes through a PKC and NF-κB-dependent mechanism. *Sci Rep* **2015**, *5*, 12442.
14. Hinterseher, I.; Schworer, C. M.; Lillvis, J. H.; Stahl, E.; Erdman, R.; Gatalica, Z.; Tromp, G.; Kuivaniemi, H. Immunohistochemical analysis of the natural killer cell cytotoxicity pathway in human abdominal aortic aneurysms. *Int J Mol Sci* **2015**, *16*, 11196–11212.
15. Prosser, D. C.; Tran, D.; Gougeon, P.-Y.; Verly, C.; Ngsee, J. K. FFAT rescues VAPA-mediated inhibition of ER-to-Golgi transport and VAPB-mediated ER aggregation. *J. Cell. Sci.* **2008**, *121*, 3052–3061.
16. Stuart, C. A.; South, M. A.; Lee, M. L.; McCurry, M. P.; Howell, M. E. A.; Ramsey, M. W.; Stone, M. H. Insulin responsiveness in metabolic syndrome after eight weeks of cycle training. *Med Sci Sports Exerc* **2013**, *45*, 2021–2029.

17. Liu, S.; Zhang, H.; Li, M.; Hu, D.; Li, C.; Ge, B.; Jin, B.; Fan, Z. Recruitment of Grb2 and SHIP1 by the ITT-like motif of TIGIT suppresses granule polarization and cytotoxicity of NK cells. *Cell Death Differ.* **2013**, *20*, 456–464.
18. Kung, M.-L.; Hsieh, C.-W.; Tai, M.-H.; Weng, C.-H.; Wu, D.-C.; Wu, W.-J.; Yeh, B.-W.; Hsieh, S.-L.; Kuo, C.-H.; Hung, H.-S.; Hsieh, S. Nanoscale characterization illustrates the cisplatin-mediated biomechanical changes of B16-F10 melanoma cells. *Phys Chem Chem Phys* **2016**, *18*, 7124–7131.
19. Fan, C.; Jiang, G.; Zhang, X.; Miao, Y.; Lin, X.; Luan, L.; Xu, Z.; Zhang, Y.; Zhao, H.; Liu, D.; Wang, E. Zbed3 contributes to malignant phenotype of lung cancer via regulating β-catenin and P120-catenin 1. *Mol. Carcinog.* **2015**, *54 Suppl 1*, E138–47.
20. Zhen, L.; Li, J.; Zhang, M.; Yang, K. MiR-10b decreases sensitivity of glioblastoma cells to radiation by targeting AKT. *Journal of Biological Research-Thessaloniki* **2016**, *23*, 357.
21. Jang, H. J.; Hong, E. M.; Park, S. W.; Byun, H. W.; Koh, D. H.; Choi, M. H.; Kae, S. H.; Lee, J. Statin induces apoptosis of human colon cancer cells and downregulation of insulin-like growth factor 1 receptor via proapoptotic ERK activation. *Oncol Lett* **2016**, *12*, 250–256.
22. Grolmusz, V. K.; Tóth, E. A.; Baghy, K.; Likó, I.; Darvasi, O.; Kovalszky, I.; Matkó, J.; Rácz, K.; Patócs, A. Fluorescence activated cell sorting followed by small RNA sequencing reveals stable microRNA expression during cell cycle progression. *BMC Genomics* **2016**, *17*, 8507.
23. Lin, G.; Brownsey, R. W.; MacLeod, K. M. Complex Regulation of PKCβ2 and PDK-1/AKT by ROCK2 in Diabetic Heart. *PLoS ONE* **2014**, *9*, e86520.
24. Fagnocchi, L.; Cherubini, A.; Hatsuda, H.; Fasciani, A.; Mazzoleni, S.; Poli, V.; Berno, V.; Rossi, R. L.; Reinbold, R.; Endele, M.; Schroeder, T.; Rocchigiani, M.; Szkarłat, Ż.; Oliviero, S.; Dalton, S.; Zippo, A. A Myc-driven self-reinforcing regulatory network maintains mouse embryonic stem cell identity. *Nat Commun* **2016**, *7*, 11903.

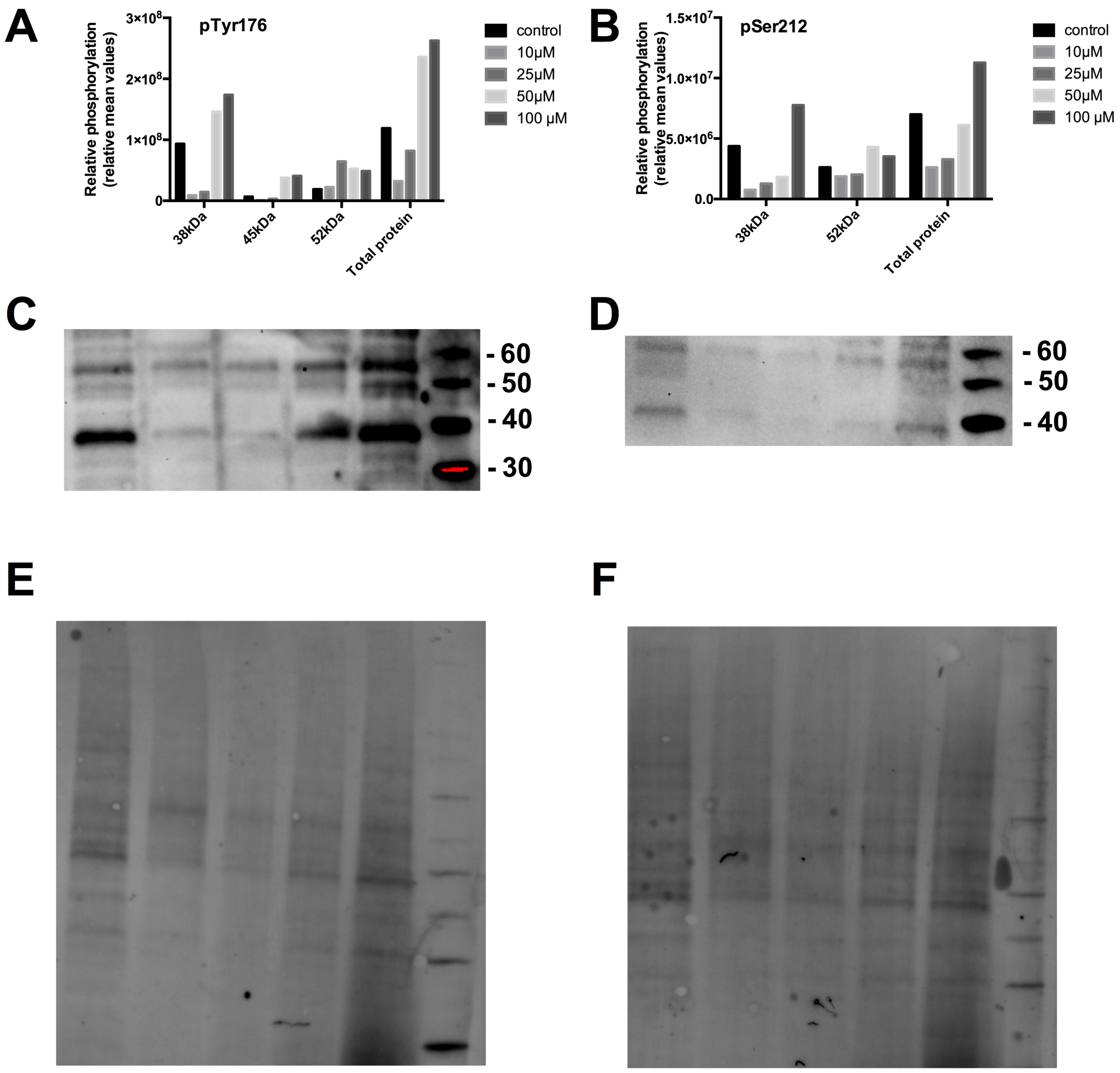

Supplementary information, Figure S1. Immunoblot analyses of the phosphotyrosine 176 and phosphoserine 212 status of PTPIP51.

A. Phosphorylation of the tyrosine 176 residue. Quantification data of the blot shown in C. Each detected isoform was quantified separately. Total protein was calculated as the sum of the three detected isoforms.
B. Phosphorylation of the serine 212 residue. Quantification data of the blot shown in D. Each detected isoform was quantified separately. Total protein was calculated as the sum of the three detected isoforms.
C. Immunoblot using the specific antibody raised against phosphorylated tyrosine 176 residue.

D. Immunoblot using the specific antibody raised against phosphorylated tyrosine 212 residue.
E. Stain-free blot for quantification of the phosphorylated tyrosine 176 residue.
F. Stain-free blot for quantification of the phosphorylated tyrosine 212 residue.

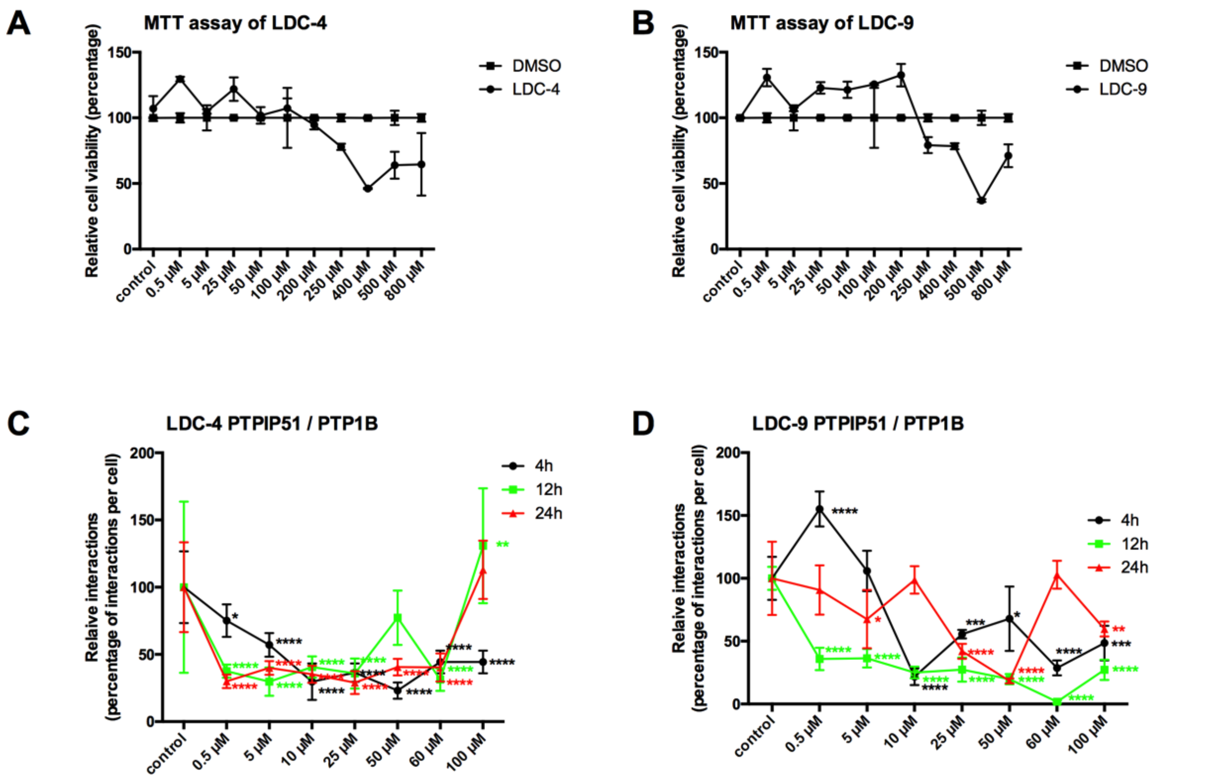

Supplementary information, Figure S2. MTT assay of HaCaT cells treated with LDC-4 and LDC-9 and the LDC-4 and LDC-9 effects on the time dependent interaction profile of PTPIP51 and PTP1B.

A. Cell viability of LDC-4 treated HaCaT cells assayed by MTT.

B. Cell viability of LDC-9 treated HaCaT cells assayed by MTT.

To exclude the toxic effect of DMSO, a second curve was established applying gradient amounts of DMSO comparable to the amount added with the rising concentrations of the effector added to the test system. The values for LDC-4 and LDC-9 treated cells were calculated as the percental quotient of the LDC-4 or LDC-9 value and the DMSO value.

C. Using LDC4 for 4 h in increasing concentrations progressively depressed the rate of PTPIP51/PTP1B interaction. Yet, highly significant differences to normal controls were only seen with concentrations starting from 10µM up to 100µM ($p<0.0001$). Extending the time of exposure to 12 h changed the pattern only at concentrations of 50µM reaching near normal values and at 100µM where the concentrations were higher than those of the controls.

Prolongation to 24h of incubation time displayed a pattern comparable to that seen after 12h of LDC4 application.

D. Application of LDC9 in increasing concentrations led to significant changes of the PTPIP51/PTP1B. After 4 h incubation the interactions slightly increased under the influence of 0.5µM LDC9 and were normalized under 5µM. Using concentrations of 10µM and higher negatively influenced the number of interactions to values being significantly different from the controls (10µM $p<0.001$, 25µM $p<0.01$, 50µM $p<0.05$, 60µM $p<0.0001$, 100µM $p<0.05$). Prolongation of the incubation time to 12h significantly reduced the number of interactions for all applied concentrations (0.5µM, 10µM, 50µM, 60µM and 100µM $p<0.0001$, 5µM and 25 µM $p<0.001$). After 24 h of incubation with LDC9 all concentrations decreased the number of interactions, but only high concentrations were able to exert a significant reduction (25µM $p<0.001$, 50µM $p<0.0001$, 100µM $p<0.05$).

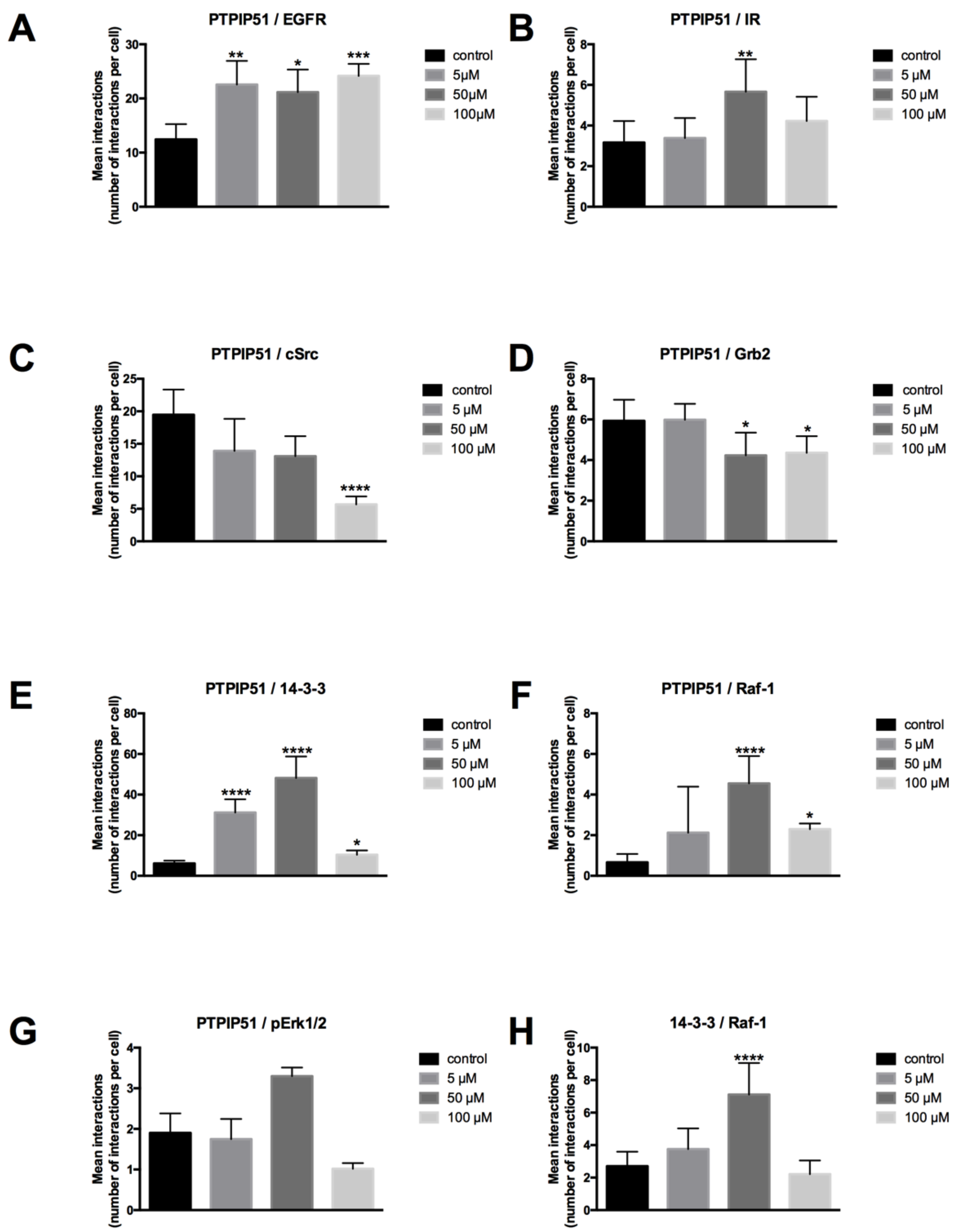

Supplementary information, Figure S3. Effects of LDC-3 on the receptor tyrosine kinase signaling and associated adapter molecules and on the MAPK signaling pathway.

Quantitative analysis of the Duolink proximity ligation assay of PTPIP51/EGFR (A), PTPIP51/IR (B), PTPIP51/c-Src (C) and PTPIP51/Grb2 (D). The interactions were evaluated

by Duolink Image Tool software in untreated controls and in cells treated with 5µM, 50µM and 100µM LDC-3 for 12h.

Quantitative analysis of the Duolink proximity ligation assay of PTPIP51/14-3-3 (E), PTPIP51/Raf-1 (F), PTPIP51/pErk1/2 (G) and 14-3-3/Raf-1 (H). The interactions were evaluated by Duolink Image Tool software in untreated controls and in cells treated with 5µM, 50µM and 100µM LDC-3 for 12h.

The resulting data were analyzed by GraphPad Prism 6 software, the significance of the results was tested by Dunnett's multiple comparisons test. * ($p < 0.05$), ** ($p < 0.01$), *** ($p < 0.001$)

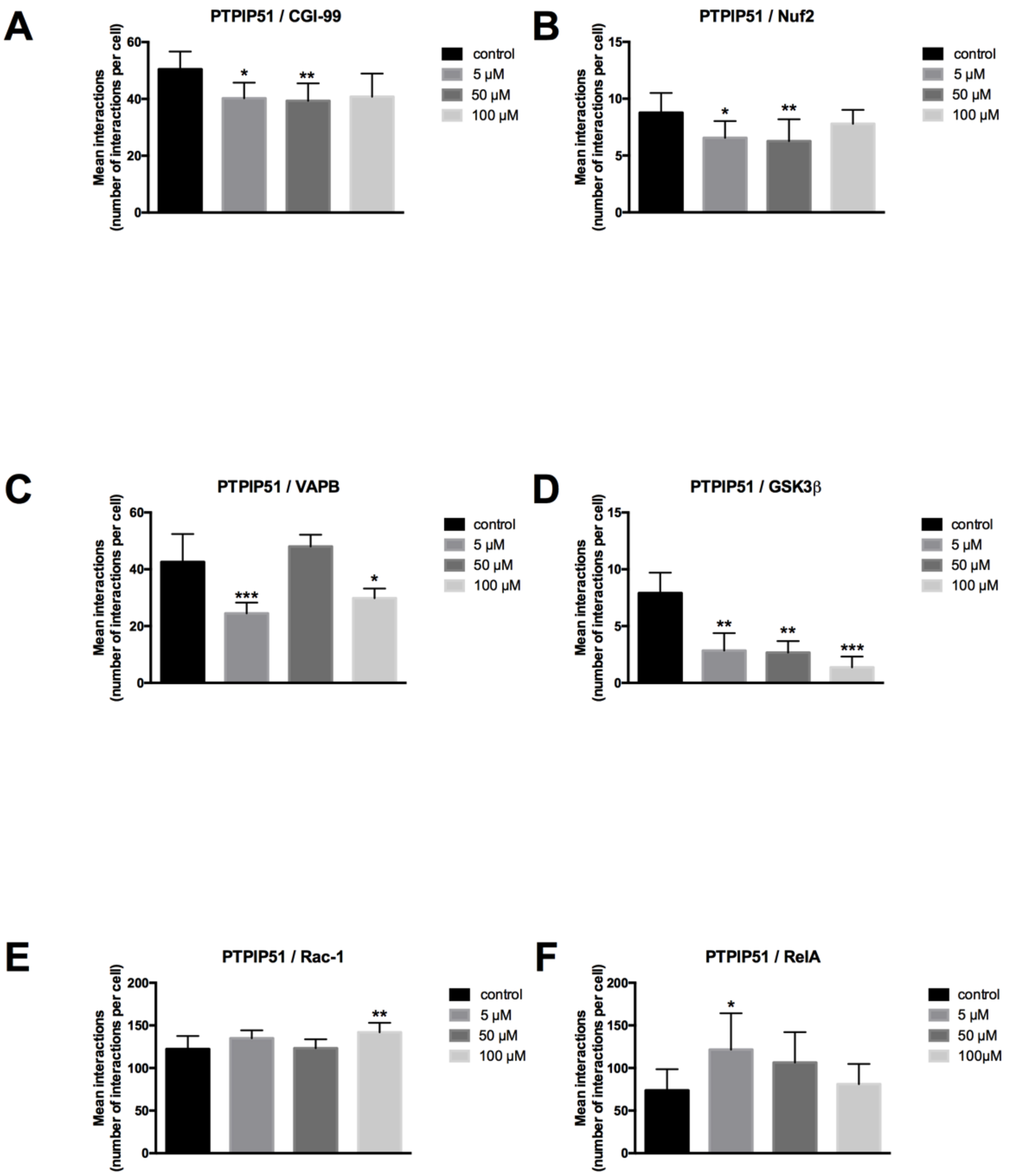

Supplementary information, Figure S4. Effects of LDC-3 on the mitotic interactome of PTPIP51, the NFkB pathway, the Ca^{2+} homestasis and the cell motility.

Quantitative analysis of the Duolink proximity ligation assay of PTPIP51/CGI-99 (A) and PTPIP51/Nuf2 (B). The interactions were evaluated by Duolink Image Tool software in untreated controls and in cells treated with 5µM, 50µM and 100µM LDC-3 for 12h.

Quantitative analysis of the Duolink proximity ligation assay of PTPIP51/VAPB (C), PTPIP51/GSK3β (D), PTPIP51/Rac-1 (E), PTPIP51/RelA (F). The interactions were evaluated by Duolink Image Tool software in untreated controls and in cells treated with 5µM, 50µM and 100µM LDC-3 for 12h.

The resulting data were analyzed by GraphPad Prism 6 software, the significance of the results was tested by Dunnett's multiple comparisons test. * ($p < 0.05$), ** ($p < 0.01$), *** ($p < 0.001$)

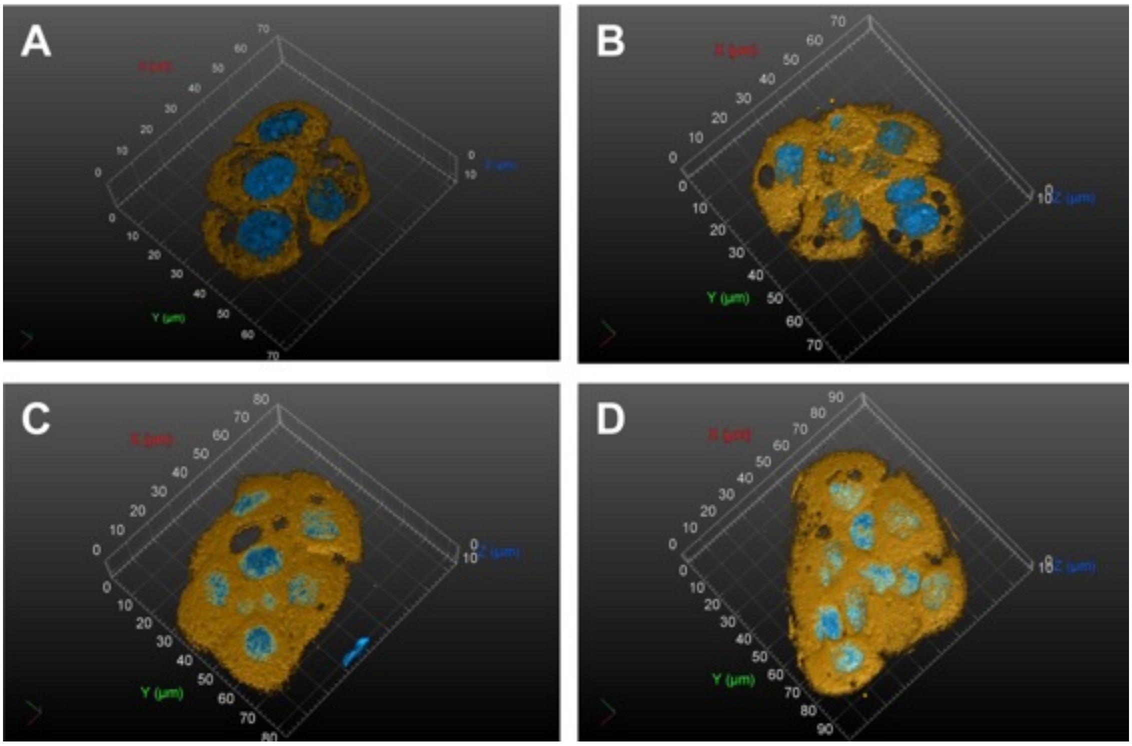

Supplementary information, Figure S5. 3D reconstruction of HaCaT cells treated with LDC-3 and immunostained with the antibody specific to phospho-Tyr176-PTPIP51.

The cells do not show any shift in the protein localization, rather than an increase in total amount of tyrosine 176 phosphorylated PTPIP51. The reconstruction was performed for the control group and each concentration of LDC-3: control (A), 5µM (B), 50µM (C), 100µM (D). The grid indicates the length in µm.

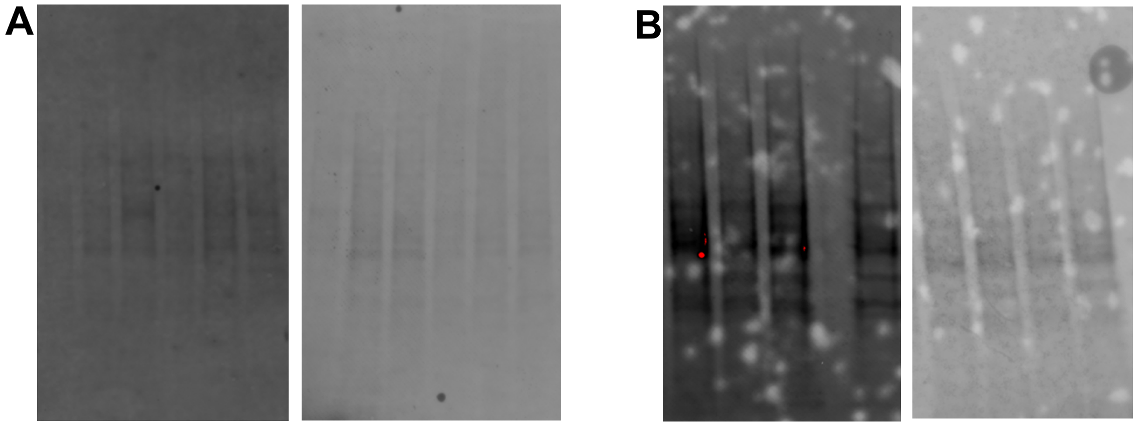

Supplementary information, Figure S6. Stain-free blot for quantification. Using this method no loading control is needed to quantify the data. (A) Stainfree-blot for the western blot analyses of phospho-MAPK and phospho-Akt (left blot) as well as of phospho-GSK3b and phospho-PKCa (right blot). (B) Stain-free blot for the siRNA knockdown experiments. Left blot: no LDC-3 treatment. Right blot: LDC-3 treatment.

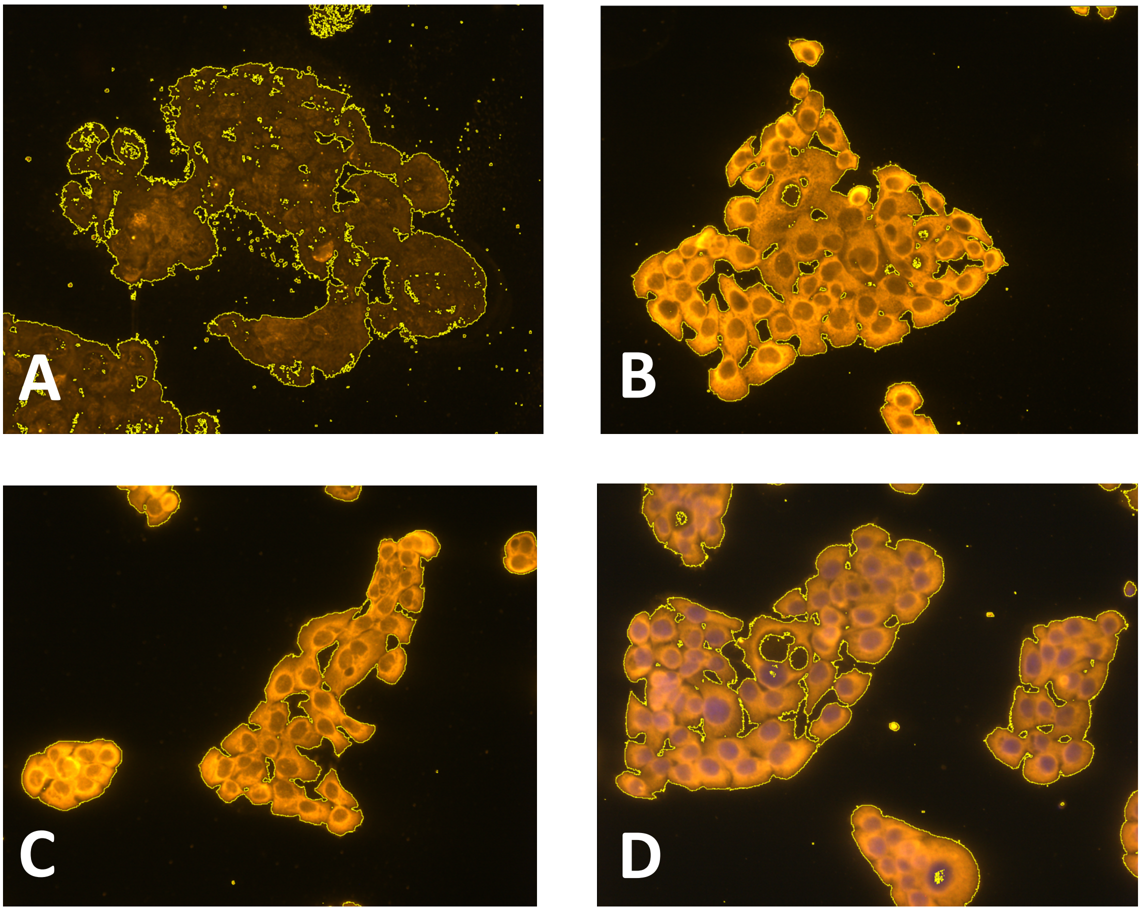

Supplementary information, Figure S7. Semiquantitative analyses of the tyrosine 176 phosphorylation status of PTPIP51. The cells were marked in ImageJ and the results of ten images (mean values of the encircled cells) were used for statistical analyses. (A) control, (B) 5µM LDC-3, (C) 50µM LDC-3, (D) 100µM LDC-3.

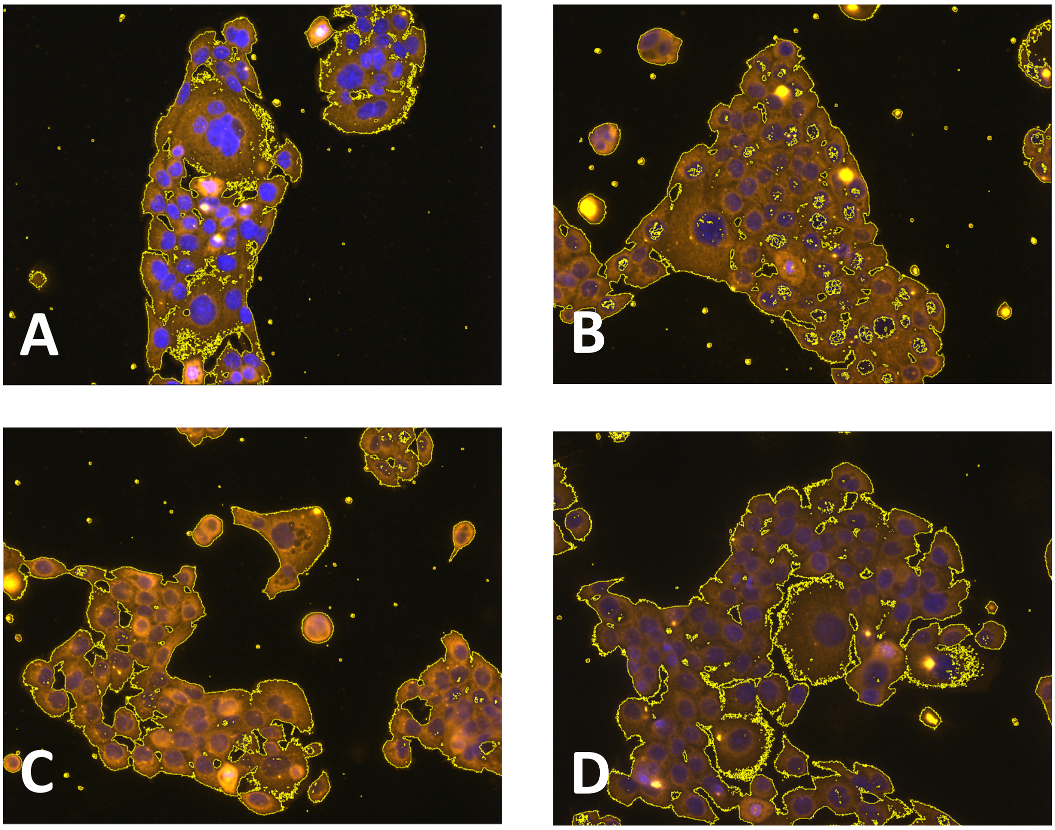

Supplementary information, Figure S8. Semiquantitative analyses of the serine 46 phosphorylation status of PTPIP51. The cells were marked in ImageJ and the results of ten images (mean values of the encircled cells) were used for statistical analyses. (A) control, (B) 5µM LDC-3, (C) 50µM LDC-3, (D) 100µM LDC-3.

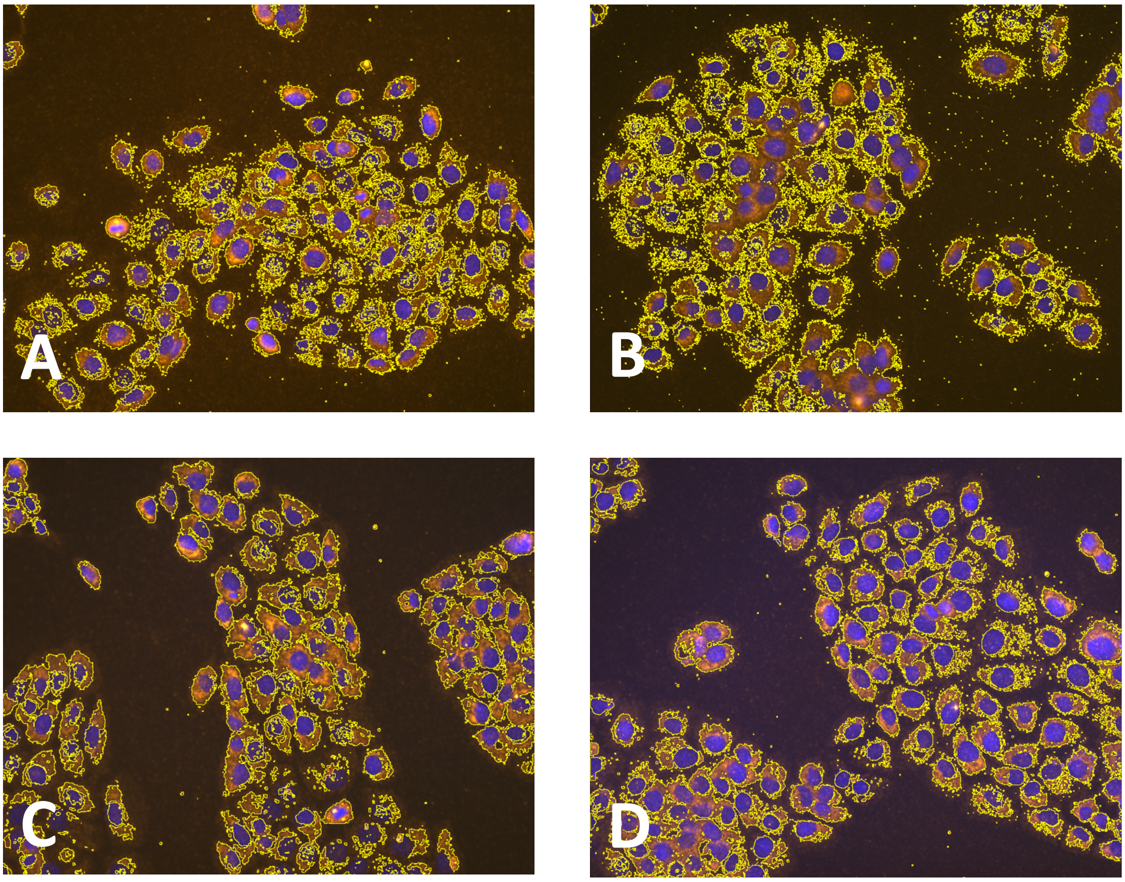

Supplementary information, Figure S9. Semiquantitative analyses of the serine 212 phosphorylation status of PTPIP51. The cells were marked in ImageJ and the results of ten images (mean values of the encircled cells) were used for statistical analyses. (A) control, (B) 5µM LDC-3, (C) 50µM LDC-3, (D) 100µM LDC-3.

<u>Semiquantitative analyses of the serine and tyrosine residues of PTPIP51</u>

phosphorylated tyrosine 176 residue of PTPIP51

control (untreated)

	Area	Mean	Min	Max
1	20.253	32.614	18	208
2	11.817	40.703	18	193
3	13.786	32.327	17	191
4	10.397	29.756	17	135
5	10.397	29.756	17	135
6	10.376	35.077	19	189
7	21.254	40.540	20	242
8	11.746	48.147	23	227
9	10.617	39.913	19	181
10	10.137	50.353	28	197

5µM LDC-3

	Area	Mean	Min	Max
1	21.316	106.514	47	198
2	21.959	108.042	48	233
3	14.200	103.601	51	204
4	9.609	117.317	50	199
5	20.017	99.299	50	181
6	15.017	111.852	48	200
7	11.216	112.814	50	184
8	7.928	119.871	47	172
9	10.572	119.908	53	177
10	16.041	121.942	45	190

50µM LDC-3

	Area	Mean	Min	Max
1	18.518	100.783	46	193
2	14.028	96.900	49	184
3	20.584	89.783	48	160
4	15.790	93.578	47	188
5	15.975	92.446	48	191
6	8.609	102.564	52	171
7	18.472	97.261	50	215
8	19.544	100.263	51	190
9	14.052	133.898	48	240
10	9.872	131.884	49	219

100µM LDC-3

	Area	Mean	Min	Max
1	18.936	90.302	49	171
2	13.985	72.923	31	160
3	8.988	78.876	43	172
4	7.788	80.493	43	196
5	11.937	78.526	38	196
6	4.702	92.515	47	202
7	11.897	84.214	44	171
8	3.306	98.974	45	194
9	12.950	96.581	44	181
10	9.946	85.396	43	181

phosphorylated serine 46 residue of PTPIP51

control (untreated)

5µM LDC-3

	Area	Mean	Min	Max
1	13.238	57.451	27	255
2	6.354	69.872	28	255
3	9.684	56.552	20	255
4	5.060	66.079	24	255
5	20.713	42.016	18	255
6	15.361	63.183	26	214
7	15.922	59.555	22	255
8	6.342	62.505	25	255
9	3.505	63.457	23	255

	Area	Mean	Min	Max
1	24.548	39.384	19	255
2	15.197	43.823	20	255
3	23.834	44.049	24	255
4	17.840	46.307	19	255
5	13.930	59.123	25	255
6	8.932	49.415	26	255
7	6.759	38.388	20	249
8	9.103	55.484	28	255
9	18.109	54.784	27	255
10	14.831	58.836	26	255

50µM LDC-3

	Area	Mean	Min	Max
1	3.957	85.812	40	255
2	7.716	69.270	27	255
3	15.006	59.534	27	255
4	6.185	64.390	27	255
5	15.225	55.365	29	255
6	14.194	57.797	24	255
7	6.181	59.167	24	255
8	7.856	45.199	20	231
9	16.518	65.125	29	255
10	5.090	80.327	30	255

100µM LDC-3

	Area	Mean	Min	Max
1	23.520	39.196	18	255
2	7.222	35.480	16	223
3	15.414	25.477	15	254
4	14.827	39.521	22	246
5	10.635	45.857	19	194
6	24.114	41.503	24	243
7	12.688	43.222	25	255
8	7.002	56.014	30	255
9	6.076	77.574	40	235
10	12.596	40.210	17	230

phosphorylated serine 212 residue of PTPIP51

control (untreated)

	Area	Mean	Min	Max
1	28.444	75.846	47	253
2	16.316	52.955	36	224
3	30.535	47.358	31	228
4	29.523	48.576	32	253

5µM LDC-3

	Area	Mean	Min	Max
1	27.216	46.313	29	253
2	25.870	44.365	29	253
3	31.511	39.094	25	253
4	25.711	44.683	27	246

5	18.545	52.662	34	198
6	24.271	49.509	34	223
7	20.051	55.668	36	191
8	18.882	55.347	37	253
9	32.889	51.089	31	253
10	20.443	59.528	35	227

5	17.937	40.963	28	214
6	24.833	40.838	29	233
7	28.079	39.163	26	253
8	12.062	48.815	31	247
9	18.222	47.780	33	124
10	26.745	40.898	27	206

50µM LDC-3

	Area	Mean	Min	Max
1	25.623	50.726	32	253
2	29.479	44.642	29	253
3	30.718	39.432	26	253
4	21.104	45.354	29	193
5	31.686	43.433	28	253
6	14.508	39.423	27	253
7	15.001	38.341	26	233
8	31.859	38.626	26	241
9	16.590	40.600	27	197
10	21.100	37.513	27	212

100µM LDC-3

	Area	Mean	Min	Max
1	30.264	49.451	32	253
2	17.462	56.793	35	184
3	17.891	53.113	32	239
4	18.292	60.607	37	244
5	29.754	54.007	35	236
6	28.200	60.935	42	253
7	26.180	57.396	36	243
8	20.222	51.877	35	253
9	27.550	48.015	32	253
10	16.252	48.988	34	253

Western Blot analysis

phospho-MAPK

	Band No.	Relative Fron	Volume (Int)	Band %	Lane %	Norm. Factor	Norm. Vol. (Int)	
Lane 1	1	0,723776	45360	100	0,976162	1	45360	control
Lane 2	1	0,724476	113352848	100	89,165007	0,880588	99817102	0,5µM LDC-3
Lane 3	1	0,725175	221391744	100	92,457641	1,620132	358683797	5µM LDC-3
Lane 4	1	0,723776	101714480	100	85,252976	2,445313	248723788	25µM LDC-3
Lane 5	1	0,718881	119398720	100	87,195497	2,399076	286446556	50µ LDC-3
Lane 6	1	0,711189	135538032	100	89,265911	2,934817	397779261	100µM LDC-3

phospho-Akt (S473)

	Band No.	Relative Fron	Volume (Int)	Band %	Lane %	Norm. Factor	Norm. Vol. (Int)	
Lane 1	1	0,429371	47269521	100	20,802828	1	47269521	control
Lane 2	1	0,421678	62230880	100	22,564182	0,880588	54799736	0,5µM LDC-3
Lane 3	1	0,420979	28790002	100	4,354193	1,620132	4664359	5µM LDC-3
Lane 4	1	0,424476	18657208	100	1,358712	2,445313	4562272	25µM LDC-3
Lane 5	1	0,421678	1145046	100	1,422396	2,399076	2747051	50µ LDC-3
Lane 6	1	0,418182	771584	100	0,779425	2,934817	2264457	100µM LDC-3

phospho-GSK3 beta

	Band No.	Relative Fron	Volume (Int)	Band %	Lane %	Norm. Factor	Norm. Vol. (Int)	
Lane 1	1	0,554882	602822	100	98,119393	1	602822	control
Lane 2	1	0,557307	6493348	100	99,635666	0,933933	6064352	0,5µM LDC-3
Lane 3	1	0,556095	6590974	100	99,605927	0,902391	5947633	5µM LDC-3
Lane 4	1	0,556095	4306780	100	98,365815	0,935498	4028982	25µM LDC-3
Lane 5	1	0,554275	31370912	100	99,865025	0,551956	17315378	50µ LDC-3
Lane 6	1	0,553062	24451868	100	99,954936	0,719338	17589162	100µM LDC-3

phospho-PKC alpha

	Band No.	Relative Fron	Volume (Int)	Band %	Lane %	Norm. Factor	Norm. Vol. (Int)	
Lane 1	1	0,478421	1057954	100	1,328877	1	1057954	control
Lane 2	1	0,476584	1470737	100	0,811643	0,933933	1373570	0,5µM LDC-3
Lane 3	1	0,471993	11184163	100	4,863087	0,902391	10092484	5µM LDC-3
Lane 4	1	0,471074	1221127	100	0,717778	0,935498	1142361	25µM LDC-3
Lane 5	1	0,471074	14976818	100	4,673779	0,551956	8266551	50µ LDC-3
Lane 6	1	0,46281	24379376	100	10,419498	0,719338	17537016	100µM LDC-3

siRNA-knockdown experiments

no LDC-3 treatement

PTPIP51

	Band No.	Relative Fron	Volume (Int)	Band %	Lane %	Norm. Factor	Norm. Vol. (Int)	
Lane 1	1	0,583567	132448000	100	73,151765	1	132448000	control
Lane 2	1	0,586368	69771500	100	50,487424	1,162271	81093387	siRNA A
Lane 3	1	0,587302	209030000	100	77,991554	0,984957	205885659	siRNA B
Lane 4	1	0,579832	114891400	100	60,225561	0,744547	85542023	siRNA C

phospho-MAPK

	Band No.	Relative Fron	Volume (Int)	Band %	Lane %	Norm. Factor	Norm. Vol. (Int)	
Lane 1	1	0,473389	115415980	100	27,06594	1	115415980	control
Lane 2	1	0,471522	79615200	100	24,249739	1,162271	92534434	siRNA A
Lane 3	1	0,467787	55238193	100	9,850461	0,984957	54407270	siRNA B
Lane 4	1	0,455649	101645160	100	19,807519	0,744547	75679577	siRNA C

LDC-3 treatement

PTPIP51

	Band No.	Relative Fron	Volume (Int)	Band %	Lane %	Norm. Factor	Norm. Vol. (Int)	
Lane 1	1	0,544563	221723635	100	74,333622	1	221723635	control

Lane 2	1	0,544563	66884769	100	45,830706	1,307511	87452553	siRNA A
Lane 3	1	0,545455	268376444	100	78,214121	1,083438	290769256	siRNA B
Lane 4	1	0,533868	90577389	100	59,933153	1,506996	136499730	siRNA C

phospho-MAPK

	Band No.	Relative Fron	Volume (Int)	Band %	Lane %	Norm. Factor	Norm. Vol. (Int)	
Lane 1	1	0,414373	325092040	100	37,457005	1	325092040	control
Lane 2	1	0,417431	166906040	100	34,044608	1,307511	218231439	siRNA A
Lane 3	1	0,420489	202981100	100	21,887193	1,083438	219917450	siRNA B
Lane 4	1	0,415138	110646760	100	29,059485	1,506996	166744184	siRNA C

Immunoblotting for the phosphorylation status of PTPIP51

Immunoblot pSer212-PTPIP51

Lane	Band No.	Relative Front	Volume (Int)	Band %	Lane %	Norm. Factor	Norm. Vol. (Int)	
1	1	0,543014	22889187	37,531729	4,000402	1	2623156	control 52kDA
1	2	0,682725	13621163	62,468271	6,658319	1	4366013	control 38 kDa
							6989169	control Total protein
2	1	0,551273	12610083	71,22605	3,042012	1,432632	1864613	10µM LDC-3 52kDa
2	2	0,68479	5851861	28,77395	1,228914	1,432632	753267	10µM LDC-3 38kDa
							2617880	10µM LDC-3 Total protein
3	1	0,559532	7778408	61,398213	2,175317	2,520859	2012515	25µM LDC-3 52kDa
3	2	0,707502	9184029	38,601787	1,367648	2,520859	1265292	25µM LDC-3 38kDa
							3277807	25µM LDC-3 Total protein
4	1	0,573297	16287023	70,27511	7,483294	1,581675	4288042	50µM LDC-3 52kDa
4	2	0,712319	15215341	29,72489	3,165276	1,581675	1813751	50µM LDC-3 38kDa
							6101793	50µM LDC-3 Total protein
5	1	0,580179	24847134	31,220198	6,10717	0,901083	3519773	100µM LDC-3 52kDa
5	2	0,721266	30967936	68,779802	13,454429	0,901083	7754253	100µM LDC-3 38kDa

							11274026	100µM LDC-3 Total protein

Immunoblot pTyr176-PTPIP51

Lane	Band No.	Relative Front	Volume (Int)	Band %	Lane %	Norm. Factor	Norm. Vol. (Int)	
1	1	0,496134	50333500	15,947454	8,446312	1	18953760	control 52kDA
1	2	0,556701	35998620	5,523725	2,925552	1	6565020	control 45 kDa
1	3	0,719072	138245520	78,528821	41,591526	1	93332540	control 38 kDa
							118851320	control Total protein
2	1	0,483247	61056800	69,678565	23,462668	0,885364	22468106	10µM LDC-3 52kDa
2	2	0,552835	5186020	2,687722	0,905029	0,885364	866665	10µM LDC-3 45kDa
2	3	0,706186	30177280	27,633713	9,305023	0,885364	8910591	10µM LDC-3 38kDa
							32245362	10µM LDC-3 Total protein
3	1	0,487113	61432280	78,572481	24,460857	2,690292	64302758	25µM LDC-3 52kDa
3	2	0,559278	5828060	3,864032	1,202934	2,690292	3162276	25µM LDC-3 45kDa
3	3	0,713918	27669600	17,563488	5,467792	2,690292	14373743	25µM LDC-3 38kDa
							81838777	25µM LDC-3 Total protein
4	1	0,488402	62225380	22,222728	13,601749	1,967189	52417834	50µM LDC-3 52kDa
4	2	0,541237	74020380	15,946085	9,760037	1,967189	37612810	50µM LDC-3 45kDa
4	3	0,706186	131115180	61,831187	37,844691	1,967189	145844241	50µM LDC-3 38kDa
							235874885	50µM LDC-3 Total protein
5	1	0,484536	109812500	18,475327	10,332215	1,384209	48541699	100µM LDC-3 52kDa
5	2	0,552835	115350900	15,457976	8,64478	1,384209	40613973	100µM LDC-3 45kDa
5	3	0,708763	212909200	66,066698	36,947404	1,384209	173582304	100µM LDC-3 38kDa
							262737976	100µM LDC-3 Total protein
